# Development of DNA Markers From Physically Mapped Loci in *Aegilops comosa* and *Aegilops umbellulata* Using Single-Gene FISH and Chromosome Sequences

**DOI:** 10.3389/fpls.2021.689031

**Published:** 2021-06-15

**Authors:** Mahmoud Said, Katerina Holušová, András Farkas, László Ivanizs, Eszter Gaál, Petr Cápal, Michael Abrouk, Mihaela M. Martis-Thiele, Balázs Kalapos, Jan Bartoš, Bernd Friebe, Jaroslav Doležel, István Molnár

**Affiliations:** ^1^Institute of Experimental Botany of the Czech Academy of Sciences, Center of the Region Haná for Biotechnological and Agricultural Research, Olomouc, Czechia; ^2^Agricultural Research Centre, Field Crops Research Institute, Cairo, Egypt; ^3^ELKH Centre for Agricultural Research, Agricultural Institute, Martonvásár, Hungary; ^4^Biological and Environmental Science and Engineering Division, Center for Desert Agriculture, King Abdullah University of Science and Technology, Thuwal, Saudi Arabia; ^5^NBIS (National Bioinformatics Infrastructure Sweden, Science for Life Laboratory), Division of Cell Biology, Department of Clinical and Experimental Medicine, Faculty of Medicine and Health Sciences, Linköping University, Linköping, Sweden; ^6^Wheat Genetics Resource Center, Kansas State University, Manhattan, KS, United States

**Keywords:** goat grasses, *Aegilops comosa*, *Aegilops umbellulata*, single-gene FISH, chromosome flow sorting and sequencing, chromosome rearrangements, homoeologous relationships, molecular markers

## Abstract

Breeding of agricultural crops adapted to climate change and resistant to diseases and pests is hindered by a limited gene pool because of domestication and thousands of years of human selection. One way to increase genetic variation is chromosome-mediated gene transfer from wild relatives by cross hybridization. In the case of wheat (*Triticum aestivum*), the species of genus *Aegilops* are a particularly attractive source of new genes and alleles. However, during the evolution of the *Aegilops* and *Triticum* genera, diversification of the D-genome lineage resulted in the formation of diploid C, M, and U genomes of *Aegilops*. The extent of structural genome alterations, which accompanied their evolution and speciation, and the shortage of molecular tools to detect *Aegilops* chromatin hamper gene transfer into wheat. To investigate the chromosome structure and help develop molecular markers with a known physical position that could improve the efficiency of the selection of desired introgressions, we developed single-gene fluorescence *in situ* hybridization (FISH) maps for M- and U-genome progenitors, *Aegilops comosa* and *Aegilops umbellulata*, respectively. Forty-three ortholog genes were located on 47 loci in *Ae. comosa* and on 52 loci in *Ae. umbellulata* using wheat cDNA probes. The results obtained showed that M-genome chromosomes preserved collinearity with those of wheat, excluding 2 and 6M containing an intrachromosomal rearrangement and paracentric inversion of 6ML, respectively. While *Ae. umbellulata* chromosomes 1, 3, and 5U maintained collinearity with wheat, structural reorganizations in 2, 4, 6, and 7U suggested a similarity with the C genome of *Aegilops markgrafii*. To develop molecular markers with exact physical positions on chromosomes of *Aegilops*, the single-gene FISH data were validated *in silico* using DNA sequence assemblies from flow-sorted M- and U-genome chromosomes. The sequence similarity search of cDNA sequences confirmed 44 out of the 47 single-gene loci in *Ae. comosa* and 40 of the 52 map positions in *Ae. umbellulata*. Polymorphic regions, thus, identified enabled the development of molecular markers, which were PCR validated using wheat-*Aegilops* disomic chromosome addition lines. The single-gene FISH-based approach allowed the development of PCR markers specific for cytogenetically mapped positions on *Aegilops* chromosomes, substituting as yet unavailable segregating map. The new knowledge and resources will support the efforts for the introgression of *Aegilops* genes into wheat and their cloning.

## Introduction

High-yielding and stress-tolerant wheat (*Triticum aestivum*, L.) cultivars with good quality traits will be needed to feed the nearly 10 billion human population estimated to be present in 2050 under changing global climate. One of the challenges is to increase genetic variation in the wheat gene pool to provide resistance to pests and diseases and tolerance to extreme abiotic conditions without significant yield loss. One way to increase the genetic variation of wheat is chromosome-mediated transfer of new gene variants from wild relatives by interspecific or intergeneric hybridization (Molnár-Láng et al., 2015).

Annual goat grasses (*Aegilops* L.) are closely related to *Triticum* and represent a rich source of genes with considerable agronomic value (Friebe et al., 1996; Schneider et al., 2008; Kilian et al., 2011). Within the genus, seven different genomes (C, D, M, N, S, T, and U) were identified in 11 diploid and 12 polyploid species (van Slageren, 1994). *Aegilops comosa* Sm in Sibth et Sm (2*n* = 2*x* = 14, MM) and *Aegilops umbellulata* Zhuk (2*n* = 2*x* = 14, UU) are diploid progenitors of the M- and U- genomes, respectively, which are also detected in 10 allopolyploid *Aegilops* species (van Slageren, 1994). *Ae. comosa* and *Ae. umbellulata* were reported as a source of resistance to wheat rust (Sears, 1956; Riley et al., 1968b; McIntosh et al., 1982; Bansal et al., 2017, 2020; Olivera et al., 2018), powdery mildew, Hessian fly, and greenbug (Riley et al., 1968a,b; Gill et al., 1985; Liu et al., 2019). With the exception of a transfer of several resistance genes from *Ae. comosa* (*Yr8, Sr34*) or *Ae. umbellulata* (*Lr9, Lr76*, and *Yr70*) (Sears, 1956; Bansal et al., 2020) to cultivated wheat (Riley et al., 1968a,b; Miller et al., 1988), their genetic diversity is largely underutilized in wheat breeding (Schneider et al., 2008).

Several factors hamper the transfer of wild alleles to wheat by interspecific hybridization. The collinearity between chromosomes of the donor species and wheat is one of them (Friebe et al., 1996). If it is broken down because of evolutionary chromosome rearrangements, homoeologous recombination may result in progenies with non-balanced genomes and poor agronomic performance (Devos et al., 1993; Zhang et al., 1998; Naranjo, 2019). Moreover, the altered structure of the donor chromosomes may interfere with recombination and compromise the attempts to reduce the size of introgressed chromatin and elimination of undesirable traits (Nasuda et al., 1998).

The efforts with chromosome-mediated gene transfer to wheat are also hampered by shortage of high-throughput techniques to screen large pre-breeding populations for the presence of alien chromatin (King et al., 2017). The most popular cytogenetic methods [genomic *in situ* hybridization (GISH) and fluorescence *in situ* hybridization (FISH)] allow the identification of M- and U-genome chromosomes or wheat-*Aegilops* intergeneric translocations (Badaeva et al., 1996a,b; Badaeva et al., 2004; Molnár et al., 2009). However, these methods are laborious and time consuming, and the tandem repeat probes frequently used for chromosome analysis using FISH in *Triticum*/*Aegilops* complexes hybridize predominantly in distal regions of chromosome arms (Molnár et al., 2011, 2016). This limits the identification of interstitial wheat-*Aegilops* translocations.

While cytogenetic analysis remains an important step in the selection procedure, marker-assisted selection would facilitate the identification of M- and U-genome chromatin at much higher throughput. This approach requires a minimum of two markers per chromosome arm, one specific for the telomeric and the other for the centromeric/near-centromeric region, so that the presence of an alien chromosome arm in the wheat background can be detected. To date, only few wheat molecular markers (AFLP, RFLP, SSR) have been tested in *Aegilops* species, and only some COS and PLUG markers were specific to *Ae. comosa* or *Ae. umbellulata* (Peil et al., 1998; Molnár et al., 2011, 2013; Liu et al., 2019). A serious problem is that the precise chromosomal location of these markers is unknown. One possibility is mapping these markers genetically, but, with *Aegilops*, it is time-consuming to develop a biparental mapping population (Zhang et al., 1998; Edae et al., 2016, 2017). Moreover, the positions of genetic markers do not always correspond to their physical locations on chromosomes, especially in the rarely recombining pericentromeric regions (Saintenac et al., 2009).

DNA sequences of conserved orthologous genes and their order are generally conserved in grass genomes (Gale and Devos, 1998), and the markers designed over the exon–intron boundaries of genes (COS markers) define orthologs regions, thus enabling the comparison of regions on the chromosomes of related species (Burt and Nicholson, 2011). In *Aegilops*, wheat COS markers were used successfully to map Quantitative trait loci (QTLs) for the B-type starch granule content and resistance genes (Howard et al., 2011). COS markers were also used to study homoeologous relationships between the M- and U-genome chromosomes of *Aegilosps* and rice, and *Brachypodium* and wheat (Molnár et al., 2013, 2016). However, the positions and the order of gene-specific markers along the chromosomes of *Aegilops* have not been determined as segregating mapping population was not available, a fact that hampers the use of the markers in marker-assisted introgression breeding.

Visualization of single-copy genes by FISH on mitotic metaphase chromosomes provides an attractive alternative to determine the exact position and the order of genes and gene-based markers along chromosomes and to investigate cross-species homology. In cereals with large and complex genomes, the use of cDNA-sequences as FISH probes allowed the visualization of single orthologous genes. Danilova et al. (2014, 2017a,b) used a set of wheat cDNA probes to develop a wheat single-gene FISH map, and analyze homoeologous relationships and chromosomal rearrangements in the tertiary gene pool of bread wheat. Single-gene FISH has also been used successfully in barley (Karafiátová et al., 2013; Danilova et al., 2017b), and *Agropyron cristatum* (Said et al., 2018) for the comparative analysis of chromosome structure and identification of gene order along chromosomes.

This study describes a new design strategy to develop molecular markers with known physical positions on chromosomes for wild gene-source species of wheat where a mapping population is not available. Using cDNA sequences as probes, we visualized and ordered a set of orthologous genes on the chromosomes of *Ae. comosa* and *Ae. umbellulata*. Furthermore, we developed draft sequence assemblies for the individual chromosomes of *Ae. comosa* and *Ae. umbellulata* and used them for the *in silico* validation of the cytogenetic positions. Using the aligned sequences of wheat and *Aegilops*, primers were designed for the mapped single-gene sequences to the regions polymorphic between *Aegilops* genomes and wheat. Finally, the primers were PCR validated in wheat, *Aegilops*, and wheat-*Aegilops* disomic addition lines representing the M- and U-genome chromosomes.

## Materials and Methods

### Plant Material

Seeds of *Ae. comosa* (2*n* = 2*x* = 14, MM) accession MvGB1039 and *Ae. umbellulata* (2*n* = 2*x* = 14, UU) accession AE740/03 were provided by the Institute of Plant Genetics and Crop Plant Research (Gatersleben, Germany) and were maintained in the Martonvásár Cereal Genebank (Martonvásár, Hungary). Seeds of bread wheat (*Triticum aestivum*) line Mv9kr1 carrying recessive crossability allele *kr1* (Molnár-Láng et al., 1996), *Aegilops biuncialis* (2*n* = 4*x* = 28, MMUU) accession MvGB642 and Mv9kr1/*Ae. biuncialis* MvGB642 amphiploid were obtained from Martonvásár Genebank (Martonvásár, Hungary). Partial sets of bread wheat (cv. Chinese Spring)/*Ae. umbellulata* (JIC2010001) chromosome addition lines 1, 2, 6, 7U, and bread wheat (cv. Chinese Spring)/*Ae. comosa* (JIC2110001) chromosome addition lines 2, 3, 4, 5, 6, and 7M were provided by Dr. Steve Reader (John Innes Centre, Norwich, UK). Bread wheat (cv. Chinese Spring)/*Aegilops geniculata* (TA2899) chromosome addition lines 3, 4, 5U^g^, and 1M^g^ (Friebe et al., 1999) were provided by Dr. Bernd Friebe (Kansas State University, Manhattan, KS, United States). Seeds of rye (*Secale cereale* L.; 2*n* = 2*x* = 14, RR) cv. Dankovské were obtained from Ms. Jarmila Kvardová (Oseva Agro, Brno, Czech Republic).

### Mitotic Chromosome Preparations

Root tip meristem cells were synchronized using hydroxyurea, accumulated in metaphase using amiprohos-methyl, and mildly fixed with formaldehyde as described by Vrána et al. (2016a,b). The synchronized root tips were used to prepare chromosome suspensions for flow cytometric sorting as described below and also for the preparation of chromosome spreads for single-gene FISH using the drop technique demonstrated by Kato et al. (2004, 2006), with modifications as described by Danilova et al. (2012) and Said et al. (2018).

### Preparation of cDNA Probes for Fluorescence *in situ* Hybridization

A total of 44 cDNA probes previously mapped by FISH to chromosomes of bread wheat (Danilova et al., 2014) and *A. cristatum* (Said et al., 2018) were used. The cDNA clones were developed by the National BioResource Project-Wheat, Japan, and all cDNA clones were provided by Dr. Tatiana V. Danilova (Department of Plant Pathology, Wheat Genetics Resource Center, Kansas State University, United States). cDNA sequences were amplified using PCR with T3/T7 primers and LongAmp DNA Polymerase (New England Biolabs, Ipswich, MA, United States) following the recommendations of the manufacturer. The PCR products were purified with the Invitrogen PCR purification kit (Life Technologies, Carlsbad, CA, United States) according to the instructions of the manufacturer.

The rye 120-bp repeat family (Bedbrook et al., 1980) was amplified by PCR from DNA clone *pSc*119.2 using M13universal primers as described by Said et al. (2018). GAA microsatellites were PCR amplified from rye (cv. Dankovské) genomic DNA with (GAA)_7_ and (CCT)_7_ primers according to Kubaláková et al. (2005), while the *Afa* family repeat was amplified from genomic DNA of bread wheat (cv. Chinese Spring) using primers AS-A and AS-B (Nagaki et al., 1995). Probes *pSc*119.2, GAA microsatellites, and *Afa* family repeat were directly labeled by PCR using aminoallyl-dUTP-CY5 (Jena Biosciences, Jena, Germany). Plasmid pTa71 (45S rDNA) (Gerlach and Bedbrook, 1979) was directly labeled by nick translation with ChromaTide® Fluorescein-12-dUTP (Thermo Fisher Scientific, Waltham, MA, United States).

All cDNA probes were labeled with ChromaTide® Texas Red®-12-dUTP (Thermo Fisher Scientific, Waltham, MA, United States). Direct labeling of cDNA and 45S rDNA probes by nick translation was done following Kato et al. (2004, 2006) and Said et al. (2018). The probe quality was checked on 1.5% agarose gel. To increase probe concentration and remove non-incorporated nucleotides, the probes were precipitated and purified by adding 173 μl 1 × TE buffer (pH 7.5), 20 μl sodium acetate (3M, pH 5.2), and 500 μl 100% ethanol; 3 μg herring sperm DNA (Promega, Madison, WI, USA) was added as blocking DNA. After overnight precipitation at −20°C, the probes were centrifuged for 30 min at 4°C at 14,000 × g, rinsed in 70% ethanol, and air dried for 7–15 min. Subsequently, the probes were dissolved in 20 μl of 2 × SSC and 1 × TE buffer (pH 7.6) in 1:1 ratio at 65°C for 10 min.

### Fluorescence *in situ* Hybridization

All FISH experiments were done with two or more probes, which were hybridized simultaneously. cDNA probes were localized in combination with 45S rDNA and one of the tandem repeats for *pSc*119.2, *Afa* family, or GAA microsatellite repeat. The experiments, such as the denaturation of probe and chromosomal DNA, hybridization and post hybridization washes, and the composition of hybridization mixture, were the same as described by Cabrera et al. (2002) and Said et al. (2018). Briefly, 300-ng cDNA probe and 50-ng DNA for each tandem repeat probe in the hybridization mixture (20 μl) were denatured at 80°C for 3 min and hybridized overnight at 37°C. After post-hybridization washes, the slides were mounted and counterstained with 4′,6-diamidino-2-phenylindole (DAPI) in a Vectashield mounting medium (Vector Laboratories, Burlingame, CA, United States).

### Microscopy, Software, Signal Capture, and Image Analysis

Chromosome preparations were examined using an Axio Imager Z.2 fluorescence microscope (Zeiss, Oberkochen, Germany) equipped with a Cool Cube 1 camera (Metasystems, Altlussheim, Germany) and appropriate filter sets. Signal capturing and picture processing were performed using the ISIS software (Metasystems, Altlussheim, Germany). Final image adjustment was done with Adobe Photoshop CS5 (Adobe Systems Incorporated, San Jose, CA, United States). Chromosome measurements and determination of the position of FISH signals were measured using the ISIS software (Metasystems).

### Chromosome Measurements

Chromosome measurements to determine the relative positions of cDNA sites were carried out as described by Said et al. (2018). Briefly, 10 best images of mitotic metaphase spreads obtained at × 100 magnification were selected and used for calculating the centromeric index, arm ratio (L/S), and relative chromosome length for each chromosome. The distance of cDNA sites from the centromere was measured in micrometers, and the average of 10 measurements was used to calculate the fraction length (FL) value. SDs and confidence intervals with a significance level of 0.05 were calculated using Microsoft Office Excel 2016 functions. The cDNA positions on wheat D-genome chromosomes and *Aegilops markgrafii* C-genome chromosomes were taken from Danilova et al. (2017a).

### Flow Cytometric Analysis and Sorting of *Aegilops* Chromosomes

The preparation of mitotic metaphase chromosome suspensions of *Ae. comosa* MvGB1039 and *Ae. umbellulata* AE740/03 was carried out as described by Vrána et al. (2000) and Kubaláková et al. (2005). Prior to the flow cytometric analysis, the chromosomes were labeled by fluorescence *in situ* hybridization in suspension (FISHIS) using 5′-FITC-GAA_7_-FITC-3′ oligonucleotides (Sigma, Saint Louis, MO, United States) according to Giorgi et al. (2013) and stained by DAPI (4′,6-diamidino 2-phenylindole) at 2 μg/ml. Chromosome analysis and sorting were carried out using a FACSAria II SORP flow cytometer and sorter (Becton Dickinson Immunocytometry Systems, San José, CA, United States) as described by Molnár et al. (2016) and Said et al. (2019). Bivariate flow karyotypes FITC vs. DAPI fluorescence were acquired for each sample and two batches of 25,000–76,000 copies of each chromosome were sorted into PCR tubes containing 40 μl sterile deionized water. The chromosome content of the flow-sorted fractions was determined by FISH on chromosomes sorted onto a microscopic slide using the probes for pSc119.2, *Afa* family repeat, and 45S rDNA according to Molnár et al. (2016). The chromosomes were classified following the karyotype described by Parisod and Badaeva (2020).

### Chromosome Sequencing and Assembly

The assembled DNA sequence contigs from flow-sorted chromosomes of *Ae. umbellulata* AE740/03 obtained previously from Bansal et al. (2020) were used for downstream analysis in this study. Chromosomes 1–7M flow sorted from *Ae. comosa* were used to prepare sequencing libraries. Samples of chromosomal DNA were purified according to Šimková et al. (2008), and 20 ng DNA was fragmented in 20 μl using Bioruptor Plus (Diagenode, Liège, Belgium) for five times for 30 s at HIGH setting. The sequencing libraries were prepared from sheared DNA using NEBNext® Ultra™ II DNA Library Prep Kit for Illumina® with the following modification: (i) size selection was directed for larger final library size (~1,000 bp) and (ii) PCR enrichment was done in nine cycles. The fragment size distribution of the libraries was checked on Agilent Bioanalyzer 2100 with High Sensitivity DNA kit (Agilent Technologies; Santa Clara, CA, United States). The samples were selected based on size using BluePippin (Sage Science, Beverly, MA, United States) in pre-cast 1.5% agarose gel cassettes. The setting of the selected range was chosen according to size distribution from Agilent Bioanalyzer results to achieve longer fragment sizes and the best recovery yield (TAB1). The recovered libraries were quantified by real-time PCR by KAPA Library Quantification Kit KK4844 (Roche, Pleasanton, CA, United States). The libraries were sequenced on an illuminaNovaSeq 6000 platform, and 2 × 150 bp paired-end reads were produced. The raw data were trimmed for low-quality bases using Trimmomatic (Bolger et al., 2014) and assembled to scaffolds with Meraculous v2.0.5 (Chapman et al., 2011) using 111-bp k-mers. Scaffolds shorter than 1 kb were eliminated. A percent of the genome represented by each chromosome is equivalent to its relative chromosome length, considering the sum of the absolute lengths (in micrometers) of all chromosomes in a haploid chromosome set (*n*) equal to 100% (Said et al., 2018). Chromosome sizes in Mbp were calculated using chromosome lengths (in micrometers) listed in Supplementary Tables 1, 2. Briefly, 1C value of a species is the amount of DNA in a haploid chromosome complement (n). For the calculation of chromosome size in Mbp, the 1C values of 5.05 and 5.53 pg for *Ae. umbellulata* and *Ae. comosa*, respectively (Furuta, 1970; Eilam et al., 2007), were used. Using the relative length of a chromosome (percent of the genome it represents) and 1C value, the amount of DNA in a chromosome can be calculated and then converted to Mbp using formula 1 pg DNA = 978 Mbp (Doležel et al., 2003). The coverage of a chromosome draft assembly is then determined by dividing the “total length of chromosome assembly (Mbp) by the chromosome size (Mbp)” value.

### Expressed Sequence Tag Mapping and Functional Annotations

Cytogenetically determined physical positions of cDNA sequences on the M- and U-genome chromosomes were validated *in silico* using a sequence similarity approach. The publicly available sequences of wheat cDNA probes (https://shigen.nig.ac.jp/wheat/komugi/) were used as queries in BLASTn searches against the reference pseudomolecules of the A, B, and D genomes of hexaploid wheat; Ensembl Plants, release-46 (Appels et al., 2018). The start position of each cDNA sequences was determined on the best hits obtained on the wheat reference sequence by the use of the BLASTn package of Blast Command Line Application 2.9.0 (ftp://ftp.ncbi.nlm.nih.gov/) with the following parameters: -task “BLASTn”; -evalue 1e-5; -max_target_seqs 2; -max_hsps 1. Translated sequences of the identified wheat genes were used for functional annotations. The Gene Ontology information was extracted from the Universal Protein Resource (ftp://ftp.uniprot.org; UniProt release 2019_11) database; the resulting protein collections were subsequently scanned with the Hidden Markov Model (HMM)-based HMMER 3.0 software package (http://eddylab.org/software/hmmer/) (Eddy, 2009) on the Pfam 32.0 entries (ftp://ftp.ebi.ac.uk) (El-Gebali et al., 2019).

Similar BLASTn searches were carried out against the chromosomal scaffolds of *Ae. comosa* MvGB1039 and *Ae. umbellulata* AE740/03, and the first two best hits were used for the *in silico* validation of single-gene FISH positions on the M- and U-genome chromosomes. The results of BLASTn, including the genomic start positions in bp on wheat chromosomes (Groups I–VII), e-value, wheat gene ID, protein domains identified in database (PFAM), and gene ontology (GO) together with the BLASTn results on chromosomal scaffolds of *Ae. comosa* and *Ae. umbellulata*, are summarized in Supplementary Data 1.

### Polymerase Chain Reaction Marker Design and Validation

To design *Aegilops* markers specific for the mapped cDNAs, pairwise alignment of wheat cDNA-sequences with the chromosomal scaffolds of *Aegilops* selected by BLASTn was carried out using UGENE software (v. 1.23). INDELs and polymorphic regions between wheat and *Aegilops* were determined, and primer pairs were designed for the exon–intron boundary and intronic regions with the Primer3 software (Untergasser et al., 2012) to amplify polymorphic fragments. At least three primer pairs per cDNA-*Ae. comosa* or cDNA-*Ae. umbellulata* alignment were designed and tested by PCR on wheat line Mv9kr1, *Ae. comosa* MvGB1039, *Ae. umbellulata* AE740/03, and *Ae. biuncialis* MvGB642. The primer pairs producing *Aegilops*-specific amplicons were further checked by PCR on Mv9kr1/*Ae. biuncialis* MvGB 642 amphiploid and partial sets of Chinese spring/*Ae. comosa*, Chinese spring*/Ae. umbellulata*, and Chinese spring*/Ae. geniculata* chromosome addition lines representing complete sets of the M and U-genome chromosomes in wheat genetic background. The reaction mixture (15 μl), containing 0.2 μmol/L of forward and reverse primers, and the thermal reaction profile used for the PCR reactions were described by Molnár et al. (2014). PCR amplicons were detected along with a 75–400 bp Range DNA Ladder using a Fragment Analyzer Automated CE System (Advanced Analytical Technologies, Ames, IA, United States) and analyzed with the PROsize v2.0 software (Advanced Analytical Technologies, Ames, IA, United States). The primer sequences, source cDNA, and *Aegilops* contigs, and the PCR amplicons obtained on different wheat and *Aegilops* genotypes are included in Supplementary Data 2.

## Results

### Chromosome Structure and Wheat-*Aegilops* Genome Relationships

**Figure 1:**
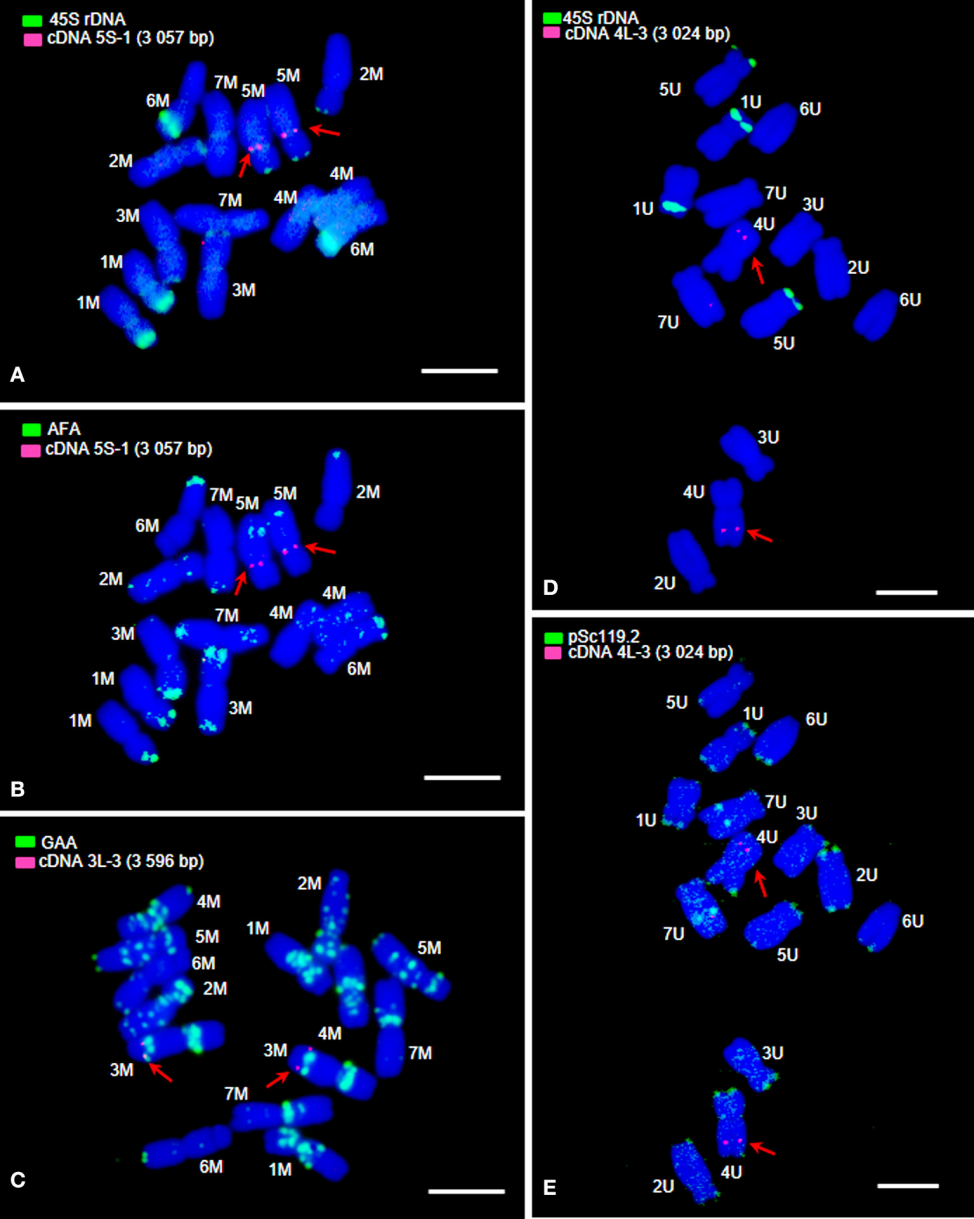
Fluorescence *in situ* hybridization (FISH) with probes for 45S rDNA, *Afa* family repeat, GAA microsatellites, *pSc*119.2 repeat, and cDNAs (red arrows) on mitotic metaphase chromosomes of *Ae. umbellulata* and *Ae. comosa*. FISH on chromosomes of *Ae. comosa* **(A)** 45S rDNA (green) and cDNA (red); **(B)** *Afa* family repeat (green) and cDNA (red); **(C)** GAA microsatellites (green) and cDNA (red). FISH on chromosomes of *Ae. umbellulata* **(D)** 45S rDNA (green) and cDNA (red); and **(E)** *pSc*119.2 repeat (green) and cDNA (red). The chromosomes were counterstained with DAPI (blue). Bars = 5 μm.

**Figure 2:**
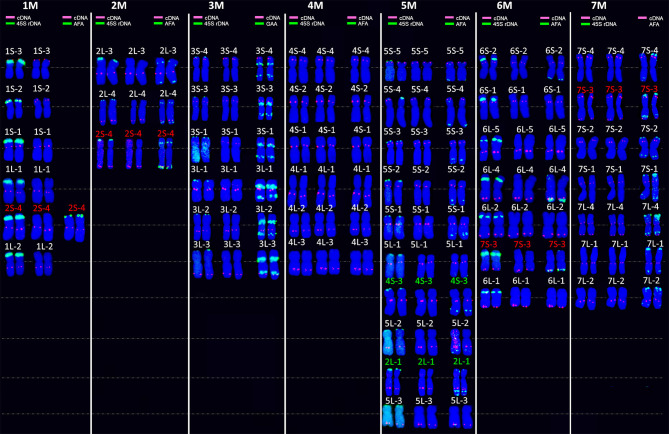
FISH on mitotic metaphase chromosomes of *Ae. comosa* reveals the distribution of 43 wheat cDNA probes (red dots), in addition to 45S rDNA, *Afa* family repeat, and GAA microsatellites (all in green). Each chromosome pair (1–7M) is shown three times, with the patterns of the repeats in addition to cDNA (left and right of the column) and with the cDNA-probes only (middle of the column). The names of the cDNA probes are shown above each chromosome pair. The names of cDNA probes that hybridized to more than one chromosome are highlighted in red. The names of cDNA-probes that hybridized only to a non-homoeologous chromosome are highlighted in green.

In order to physically map single orthologous genes to chromosomes, we used FISH with cDNA-probes in combination with probes for DNA repeats, whose hybridizing patterns on the M- and U-genome chromosomes are known (Molnár et al., 2016; Parisod and Badaeva, 2020). 45S rDNA, *Afa* family repeat, and GAA microsatellites permitted the identification of the whole chromosome complement of *Ae. comosa*, while 45S rDNA and *pSc*119.2 repeats were used to identify the chromosomes of *Ae. umbellulata* (Figures 1, 2, Supplementary Figures 1–3). The hybridization patterns together with the data on chromosome size and centromere position are summarized in Supplementary Figures 1–3 and Supplementary Tables 1, 2.

**Figure 3:**
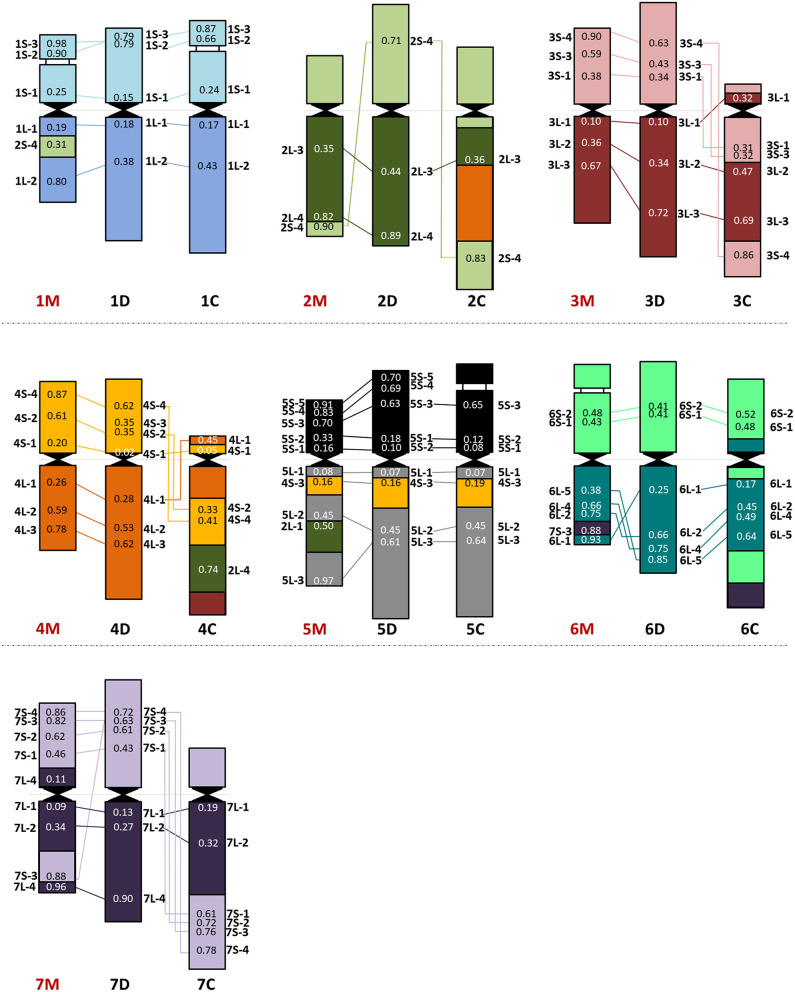
Chromosome organization of the *Ae. comosa* M genome **(left)** compared with the D (sub)genome of wheat **(middle)** and the *Ae. markgrafii* C genome **(right)**. The average relative positions of cDNAs are shown on the chromosomes.

A set of 44 wheat cDNA-probes covering wheat homoeologous groups 1–7 (4–8 cDNA probes per chromosome) was used for FISH to investigate the long-range organization of the M- and U-genome chromosomes and their homology with those of hexaploid wheat and *Ae. markgrafii*. One cDNA sequence (5L-4) failed to produce PCR amplicon and was discarded from the FISH experiments. The remaining 43 cDNA-probes produced clear hybridization signals in 47 positions on the chromosomes of *Ae. comosa*. Thirty-nine probes (90.7%) were hybridized to the same homoeologous chromosome groups as in wheat at a total of 40 loci. Some cDNAs were located at two (2S-4 and 7L-4) or three different positions (7S-3) on chromosomes homoeologous and non-homoeologous with wheat. Finally, two (4.7%) cDNA probes (4S-3 and 2L-1) were detected only on single loci on non-homoeologous M-genome chromosomes relative to wheat (Figures 2, 3, Supplementary Table 3).

In general, chromosomes 1, 3, 4, 5, and 7M of *Ae. comosa* were found collinear with the corresponding wheat chromosomes. However, a collinearity distortion on chromosome 2M was indicated by wheat group 2 short arm subtelomeric-specific cDNA-probe 2S-4, which localized to the telomeric regions of the 2M long arm and interstitial region of 1ML, and by group 2 long arm-specific marker located on 5ML. A paracentric inversion was detected on 6ML relative to wheat (Figure 3). The hybridization sites of two group 7–specific probes were also duplicated (7L-4) or triplicated (7S-3).

**Figure 4:**
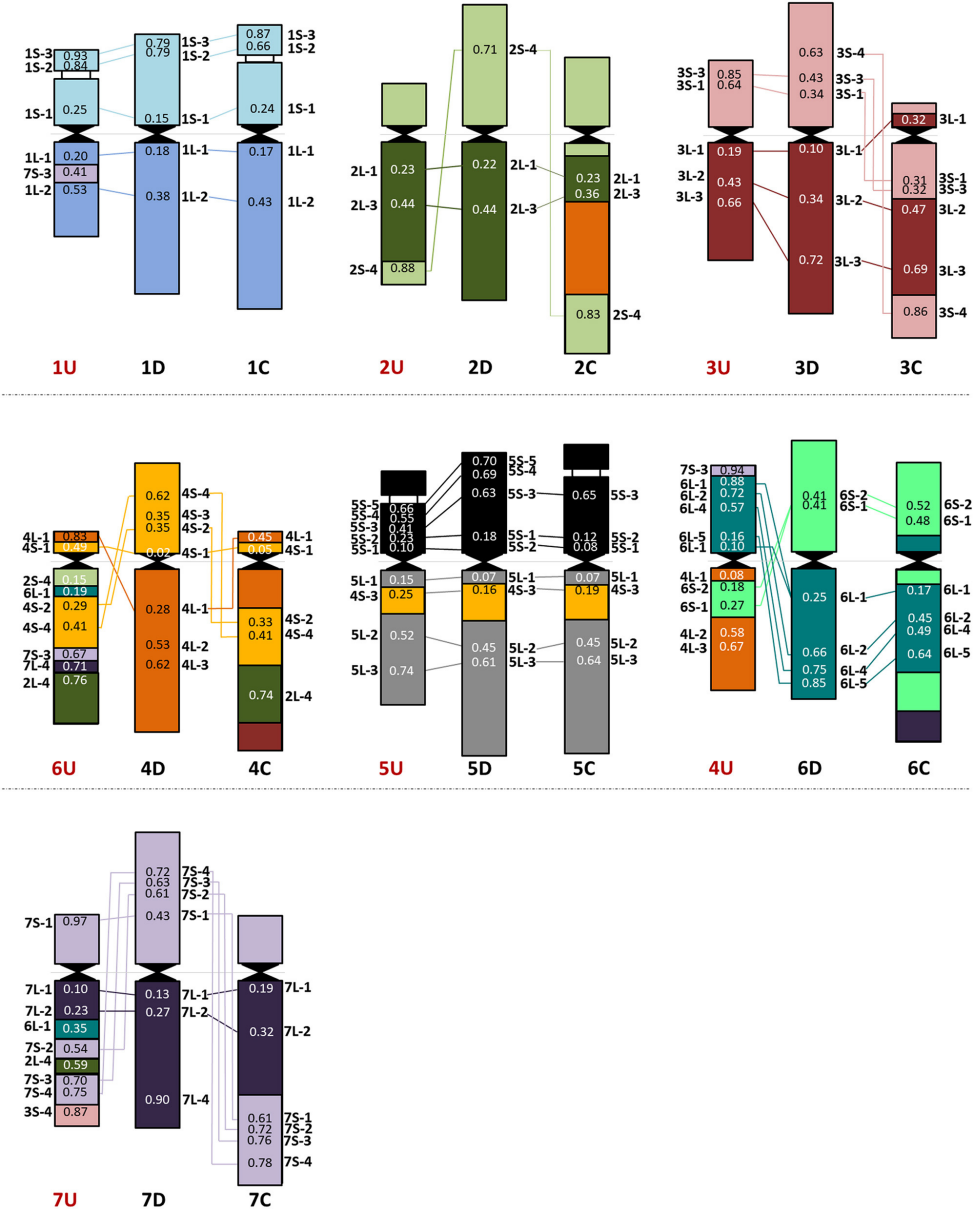
Chromosome organization of the *Ae. umbellulata* U genome **(left)** compared with the D (sub)genome of wheat **(middle)** and the *Ae. markgrafii* C genome **(right)**. The average relative positions of cDNAs are shown on the chromosomes.

The 43 wheat cDNA-probes hybridized at 52 loci in *Ae. umbellulata* and the positions of these hybridization signals indicated significant genome rearrangements in U-genome relative to wheat (Figure 4, Supplementary Figure 3, Supplementary Table 4). Apart from the signal of 7S-3 on 1UL, chromosomes 1, 3, and 5U (excluding the telomeric part of the 3U short arm which was detected in distal position of 7UL) were generally collinear with the corresponding wheat chromosomes. No probes produced signals on 2US as the cDNA-probes specific for this chromosome arm were detected on telomeres of 2UL and 6UL. The probe specific for the distal part of the wheat group 2 long arm (2L-4) also hybridized on 6UL and 7UL. The most pronounced collinearity distortion relative to wheat was observed for chromosomes 4, 6, and 7U. For example, the signals of group 4-specific cDNA-probes on 6U and group 6 probes on 4U indicated multiple reciprocal translocations between chromosomes 4 and 6U together with inversion on 4US. Chromosome 6U exhibited the most rearranged structure, as its short arm showed homology with wheat group 4, while its long arm contained regions homoeologous with group 2, the short arm of group 4, long arm of group 6, group 7, and pericentric inversion. Finally, an intra-chromosomal translocation on 7U relative to wheat was detected with a putative breakpoint between 7US-1 and 7US-2 on the short arm, and translocations of the distal half of the short arm to the end of the long arm accompanied/followed by inversion of the interstitial region of 7UL and further insertions of fragments from groups 2, 3, and 6.

### *In silico* Validation of cDNA Locations Using Sequences From Flow-Sorted Chromosomes

**Figure 5:**
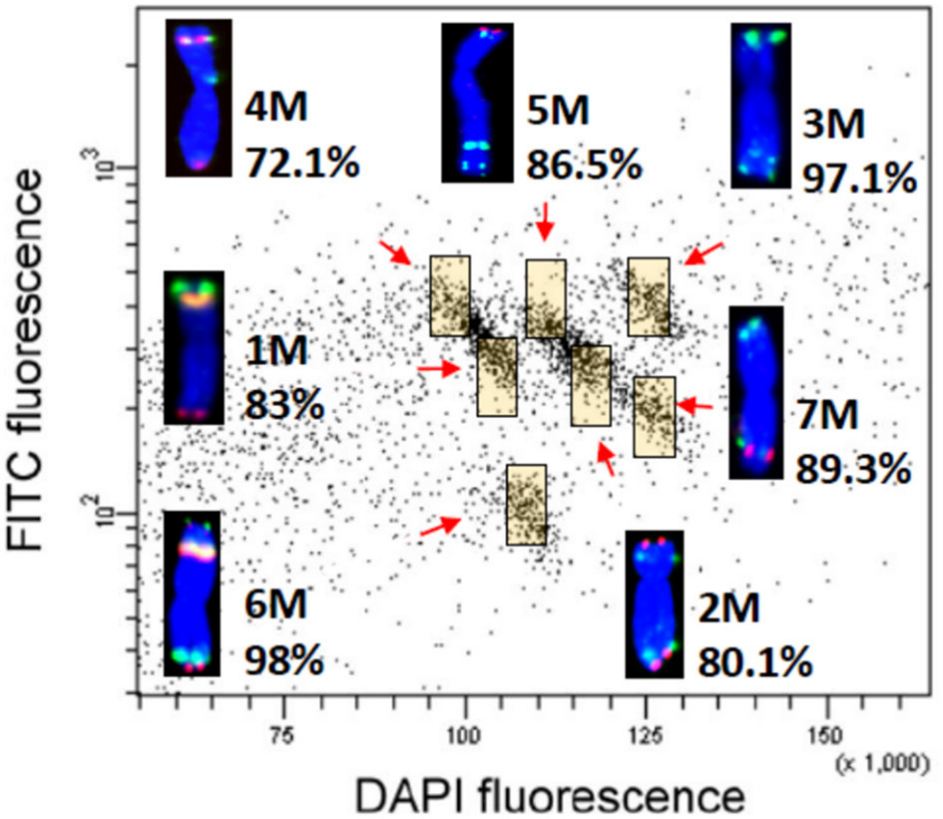
Bivariate flow karyotyping of *Ae. comosa* chromosomes after fluorescence *in situ* hybridization in suspension (FISHIS) with probes for (GAA)_7_ and (ACG)_5_ resolved all seven M-genome chromosomes, which could be flow-sorted at purities of 72–98%. Chromosomes were assigned to the colored regions (yellow) by fluorescence *in situ* hybridization using probes for 45S rDNA (yellow), *Afa* family (green), and pSc119.2 (red). Chromosomes were counterstained by DAPI (blue).

The cytogenetically determined positions of wheat single-gene (cDNA) probes were validated by sequence similarity search of cDNA sequences against the chromosomal assemblies of *Ae. comosa* and *Ae. umbellulata*. In the case of *Ae. umbellulata*, previously developed chromosome assemblies were used for the analysis. In order to obtain chromosome assemblies for *Ae. comosa*, each of the M-genome chromosomes was sorted by flow cytometry and then shot-gun sequenced. Bivariate flow karyotypes GAA-FITC vs. DAPI fluorescence comprised three clearly separated and two mixed populations (Figure 5). FISH with chromosomes sorted onto a microscope slide (Figure 5) showed that the well-resolved populations corresponded to pure (89–98%) fractions of chromosomes 3, 6, and 7M (Supplementary Table 5), while chromosomes 1 and 4M and 2 and 5M could be sorted from the two mixed populations at 72–86% purities. A total of 67,000–150,000 chromosomes were sorted to prepare 59.5 ng DNA on average from each chromosome (Supplementary Table 5). Shotgun sequencing of chromosomal DNA resulted in 112.44–210.01 Gb output for each sample (Table 1). From 52% (5M) to 89% (4M), the chromosome sizes were represented in the total length of the draft assemblies. The statistical parameters of the chromosome assemblies are summarized in Table 1.

**Table 1:** Sequencing and assembly statistics of *Ae. comosa* and *Ae. umbellulata* chromosomes.

**Source genotype**	**Chromosome**	**Output [Gb]**	**Scaffolds**	**Total length of assembly [Mb]**	Chromosome size^*^ (Mb)	**Assembly coverage**	**Max scaffolds size [kb]**	**N50**	**N50 length [kb]**
*Ae. comosa* MvGB1039	1M	149.86	65 680	437.1	792.36	0.55	309.7	8 494	14.4
	2M	210.01	186 366	813.7	920.08	0.88	139.6	35 199	6.4
	3M	123.04	61 390	534.4	944.26	0.57	174.5	8 478	18.3
	4M	122.38	157 754	736.7	823.98	0.89	309.5	27 632	7.1
	5M	112.44	63 499	481.6	931.24	0.52	179.4	9 611	14.5
	6M	157.27	49 989	463.2	854.36	0.54	221.6	6 584	20.2
	7M	132.35	59 000	531.4	933.10	0.57	309.7	8 192	18.9
*Ae. umbellulata* AE740/03	1U	19.98	136 919	300.0	649.23	0.46	44.6	24 762	3.2
	2U	46.83	190 616	382.9	718.08	0.53	50.8	38 317	2.9
	3U	28.57	157 796	352.7	700.74	0.50	43.7	27 182	3.4
	4U	26.83	115 777	264.5	814.47	0.32	48.5	23 092	3.2
	5U	30.50	101 001	234.7	789.99	0.3	46.8	19 505	3.3
	6U	19.79	130 057	324.5	679.83	0.48	56.4	21 333	4.0
	7U	26.67	145 222	388.7	747.66	0.52	63.7	29 764	3.5

* *Chromosome size calculated from the micrometer data presented in Supplementary Tables 1–2. The total length of the whole chromosome complement in the haploid set (1–7M; 1–7U) corresponds to the 1C value (DNA amount in pg of the haploid genome) of the species. The relative length of each chromosomes was converted into pg DNA values, which were further converted into Mb values using equation 1pg DNA = 978 Mbp described by Doležel et al. (2003)*.

**Figure 6:**
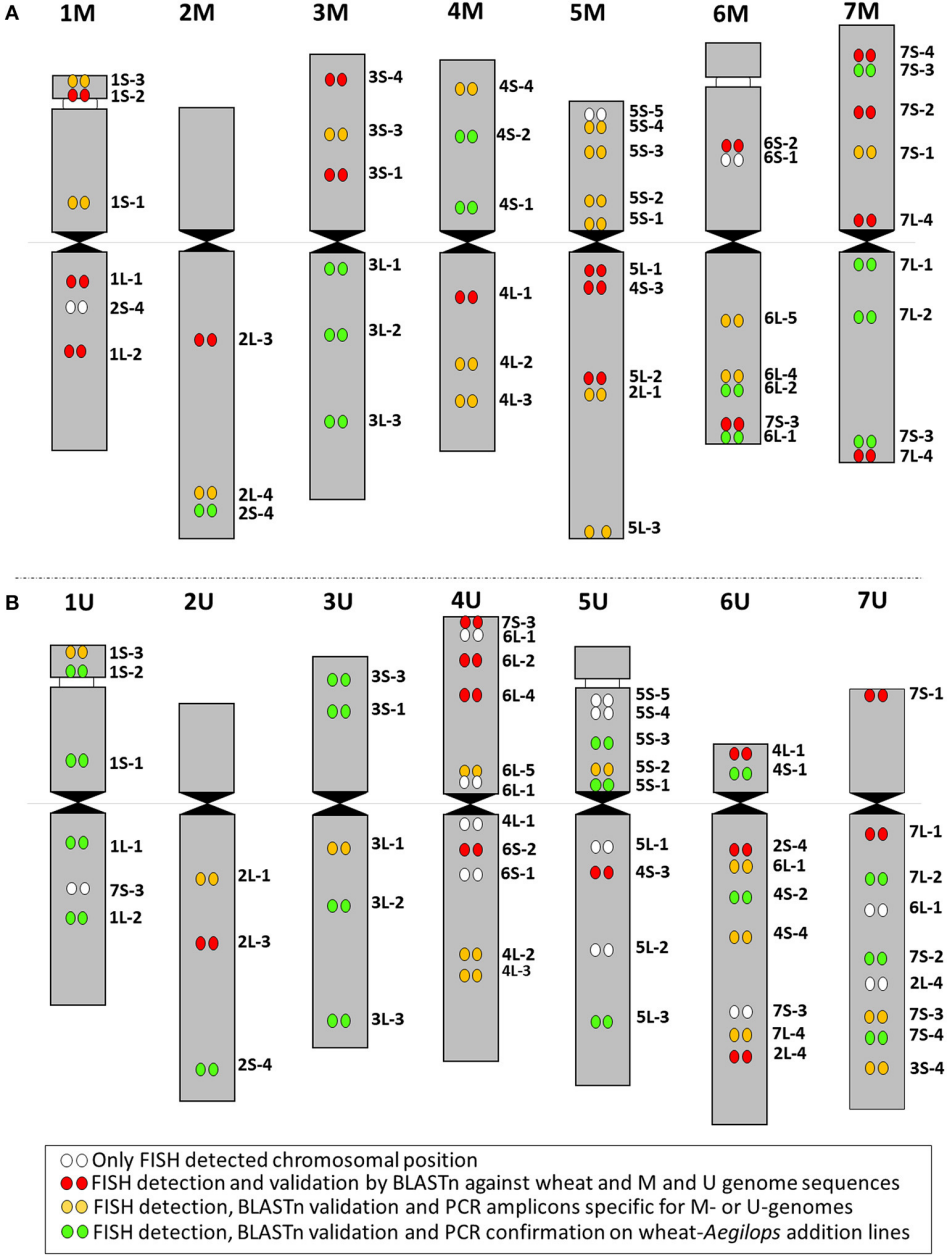
Idiogram summarizing the results of FISH mapping of cDNAs on **(A)** M- and **(B)** U-genome chromosomes. The color scheme (bottom) indicates detection of the cDNAs as FISH probes on M and U genomes (white dots), detection as FISH probes and *in silico* validation by BLASTn against bread wheat and M- and U-genome sequences (red dots); detection as FISH probes, validation by BLASTn and PCR amplicons specific for M or U genomes (yellow dots); detection as FISH probes, validation by BLASTn, and PCR markers validated on wheat-*Aegilops* addition lines (green dots).

The 44 cDNA query sequences resulted in 44 on the genome assemblies of wheat, while 88 and 86 significant hits on the chromosome assemblies of *Ae. comosa* and *Ae. umbellulata*, respectively, as we used the first two best hits for the analysis (Supplementary Data 1). The generally similar alignment length of the cDNA-sequences on wheat and on the two *Aegilops* species and the high identity of the aligned sequences (mean value: >95%) in the three species reflected high homology in coding regions between wheat and the *Aegilops* species. Finally, out of the 47 cytogenetically determined single-gene positions on *Ae. comosa* chromosomes, 44 (93.6%) were confirmed when one of the two best hits of the cDNA-sequences was found in the assembly of the same chromosomes as determined by FISH (Figure 6A). Similarly, 40 (76.9%) out of the 52 single-gene FISH sites of the *Ae. umbellulata* map were confirmed (Figure 6B).

### Marker Design and Validation

**Figure 7:**
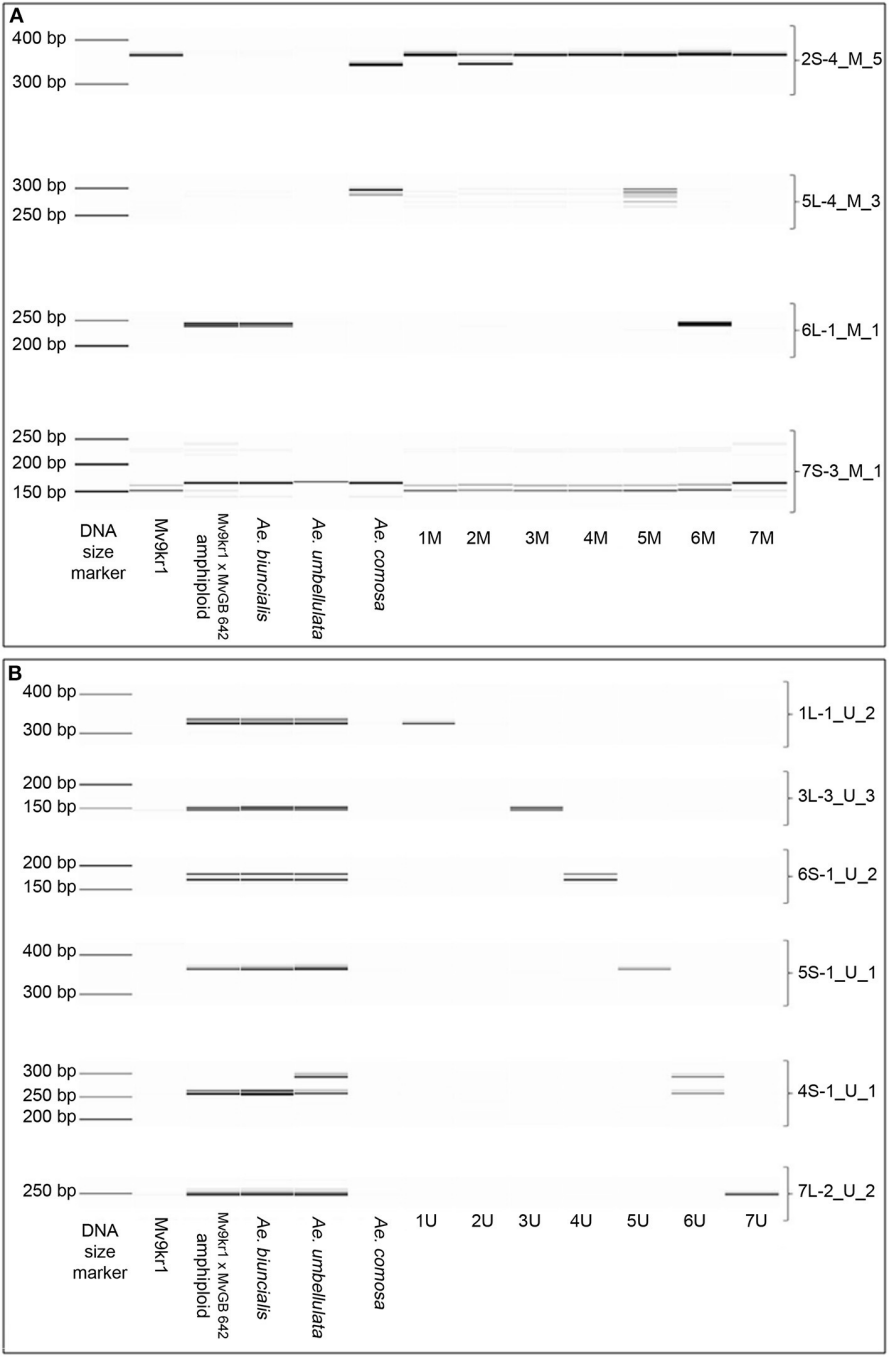
Digital capillary electrophoretic pattern of the cDNA-derived markers designed by the use of corresponding **(A)** M or **(B)** U chromosomal sequences determined by the BLASTn search. The markers were tested on hexaploid wheat line Mv9kr1, on *Ae. biuncialis* MvGB642 accession, wheat-*Ae. biuncialis* MvGB 642 amphiploid, and *Ae. comosa* MvGB1039 and *Ae. umbellulata* AE740/03. The markers were also tested on wheat-*Ae. comosa* (2M−7M), wheat-*Ae. umbellulata* (1U, 2U, 6U, 7U), and wheat- *Ae. geniculata* (1M, 3U, 4U, 5U) disomic chromosome addition lines representing the whole set of M- or U-genome chromosomes. A 35–500 bp DNA ladder was used as a molecular-weight size marker to estimate the fragment size.

In order to design PCR markers specific for the cytogenetically mapped single genes, 44 full-length cDNA sequences were aligned to the corresponding *Aegilops* contigs (Supplementary Data 1) and 274 primer pairs (136 and 138 primer pairs using M- and U-contigs, respectively) were designed specifically for the exon–intron boundaries and the intronic region (Supplementary Table 6, Supplementary Data 2). The markers polymorphic between the M- and U- genomes of *Aegilops* and wheat were validated by PCR using genomic DNA from hexaploid wheat (Mv9kr1), *Ae. comosa* (MvGB1039), *Ae. umbellulata* (AE740/03), and allotetraploid (M^b^M^b^U^b^U^b^) *Ae. biuncialis* (MvGB642) as the template (Figure 7, Supplementary Data 2).

Out of the 136M-genome markers, 68 were polymorphic between *Aegilops* and wheat (16 and 52 showed presence/absence and size polymorphism, respectively) (Figure 7A, Supplementary Table 6, Supplementary Data 2). In the case of the 138 U-genome markers, 74 were polymorphic between *Aegilops* and wheat (37 and 37 were presence/absence and size polymorphic, respectively) (Figure 7B, Supplementary Table 6, Supplementary Data 2). Independent of which *Aegilops* genomic contig was used for marker design, 32 markers amplified a specific PCR product from both the M- and U-genomes (Supplementary Data 2). Finally, 34 markers were M-genome specific (produced amplicons only from *Ae. comosa* and *Ae. biuncialis* which were polymorphic for wheat), and 48 were U-genome specific (produced amplicons only from *Ae. umbellulata* and *Ae. biuncialis* which were polymorphic relative to wheat) (Supplementary Data 2).

The 66 markers producing amplicons from the M genome (34 M-genome specific and 32 M-/U-genome specific) were related to 27 BLAST validated single-gene positions on chromosomes 1–7M of *Ae. comosa*. In the case of *Ae. umbellulata*, the 80 markers producing amplicons from the U-genome (48 U-genome specific and 32 M-/U-genome specific) were related to 28 BLAST-validated cDNA sites on all of the chromosomes, excluding chromosome arms 2US and 7US. The marker coverage of the chromosomes ranged from three (2U) to six (7U) (Figures 6B, 7B).

The PCR markers (at least one marker per cDNA site) were further validated on wheat-*Aegilops* addition lines carrying chromosomes 1–7M and 1–7U. To cover the 27 cDNA sites of M-genome, 28 randomly selected markers were tested. In total, 11 markers confirmed 12 single-gene positions (44.4). Moreover, cDNA-probe 5L-4, whose location by FISH failed, was also located on chromosome 5M. In total, PCR markers were validated on the addition lines for one gene position on chromosome 2M, for two on 4 and 6M, for three on 3M, and for four positions on 7M (Figure 6A, Supplementary Data 2).

We tested a total of 34 randomly selected markers for the 28 cDNA sites of the U-genome. Out of the 30 markers with *Aegilops*-specific amplicons covering 24 cDNA sites, 21 markers confirmed 17 single gene positions (60.7%), as they were assigned to the same U-genome chromosomes, as determined by single-gene FISH and by BLASTn. The PCR markers were validated on the addition lines for one cDNA position on 2U, for two on 6U, for three on 5U and 7U, and for four gene positions on 1U and 3U (Figure 6B, Supplementary Data 2). A PCR marker for cDNA-sequence 5L-4 was located on chromosome 4U.

## Discussion

The single-gene FISH maps developed during the course of this study significantly improve the knowledge on the long-range chromosome organization in *Ae. comosa* and *Ae. umbellulata*. The new results and molecular tools will make the alien gene transfer from these species into wheat more efficient.

As the 44 orthologous genes used in this study were also mapped on the A, B, and D subgenomes of hexaploid wheat (Danilova et al., 2014), on C-genome chromosomes of *Ae. markgrafii* (Danilova et al., 2017a), the single-gene FISH maps of *Ae. comosa* and *Ae. umbellulata* provide a unique opportunity to investigate the karyotype evolution in the *Triticum*/*Aegilops* complex. The results support the previous observations obtained by molecular markers specific for conserved orthologous genes, suggesting a more similar organization of the M genome of *Ae. comosa* than those of the U-genome chromosomes of *Ae. umbellulata* relative to wheat (Molnár et al., 2016; Liu et al., 2019). However, as shown in the previous studies, the marker positions were not determined along the chromosomes of *Aegilops*, and the identification of intrachromosomal rearrangements was not possible. We observed a well-preserved wheat-*Ae. comosa* collinearity for chromosomes 1, 3, and 4M and, to a lesser extent, for chromosomes 5 and 7M (Figures 2, 3, Supplementary Table 7), while conserved wheat-*Aegilops* synteny was observed only for chromosomes 1, 3, and 5U in *Ae. umbellulata* (Figure 4, Supplementary Figure 3, Supplementary Table 7).

In agreement with Nasuda et al. (1998), who identified terminal intra-chromosomal translocation in chromosome 2M relative to wheat group 2, we detected a distal part of the short arm on the distal end of group 2 long arms in *Ae. comosa* as well as in *Ae. umbellulata*. This translocation was also detected in *Ae. umbellulata* by genetic mapping (Zhang et al., 1998; Edae et al., 2017) and in *Ae. markgrafii* by single-gene FISH (Danilova et al., 2017a). However, the break points on chromosome group 2 long arms were different in the three species as most distal cDNA-probe 2L-4 was present on 2ML, while this region was translocated to the distal part of 6UL, or to 4CL. Similar organization of chromosomes 6U and 4C was also highlighted by the pericentric inversion of group 4 short and long arm regions. Another example of a similar genome structure of *Ae. umbellulata* and *Ae. markgrafii* was the intrachromosomal translocation detected in chromosomes 7U and 7C, where the chromosome region homoeologous to the group 7 short arm of wheat was translocated to the long arm in *Ae. umbellulata* and *Ae. markgrafii* accompanied/followed by paracentric inversions on 7UL and 7CL. However, this rearrangement was not detected in *Ae. comosa* (Figures 2–4, Supplementary Figure 3).

The wheat-*Aegilops* collinearity was also interrupted on the chromosome arm of 6ML because of paracentric inversion relative to wheat (Danilova et al., 2014). In fact, a similar inversion of the wheat group 6 long arm was observed in *Ae. umbellulata*, where the inverted region was translocated to 4US, in diploid and tetraploid *A. cristatum* (Han et al., 2014; Said et al., 2018), but it was not found in *Ae. markgrafii* (Danilova et al., 2017a). Chromosomes 4U and 6U showed the most rearranged structure in *Ae. umbellulata*. The localization of four out of the seven group 4-specific cDNA-probes on 6U and all of the six group 6-specific probes on 4U indicated multiple reciprocal translocations between 4U and 6U, confirming a need to rename these chromosomes as recommended by Zhang et al. (1998). Finally, the detection of a cDNA probe from the wheat group 3 short arm on 7UL indicated another interchromosomal translocation in *Ae. umbellulata* (Figures 2–4, Supplementary Figure 3).

The comparative single-gene FISH analysis highlighted the role of karyotype changes in the evolution of closely related C-, M- and U-genomes of *Aegilops*. Recent phylogenomic studies on diploid *Triticum*/*Aegilops* species using 38 nuclear low copy loci (Huynh et al., 2019) and by genome-wide RNA-seq-based polymorphic analysis (Glémin et al., 2019; Tanaka et al., 2020) have shown that the ancestor of the D-genome lineage went through intensive diversification ~3 Mya, leading to the formation of the current *Aegilops* species with C, D, M, N, S, and U genomes in the area of Fertile Crescent and their subsequent radiation to the Mediterranean landscapes (Kilian et al., 2011). Glémin et al. (2019) and Tanaka et al. (2020) suggested that C-, M-, N-, and U-genome species formed a separated group from those containing the D and S genomes. The C genome of *Ae. markgrafii* and U-genome of *Ae. umbellulata* were found to be closely related, forming a clade separated from the species containing the M genome of *Ae. comosa* and the N genome of *Ae. uniaristata* (Glémin et al., 2019; Tanaka et al., 2020). Consistent with these results, the single-gene FISH map of *Ae. comosa* and *Ae. umbellulata* together with those of *Ae. markgrafii* (Danilova et al., 2017a) showed that the organization of the M genome remained relatively similar to the three subgenomes of bread wheat (Figures 2, 3). On the contrary, highly rearranged C and U genomes, which are similar to each other, are significantly different from *Ae. comosa* as reflected by the similar alterations of the structure of chromosomes 4C and 6U (pericentric inversion of group 4 short and long arm regions, the presence of group 2 long arm region on the long arms) and 7C and 7U (intra-chromosomal translocation of the distal half of short arm to the long arm accompanied/followed by paracentric inversions on 7CL and 7UL) (Figure 4, Supplementary Figure 3).

Chromosome rearrangements could be the outcome of introgressive hybridization with an unknown species, as it was suggested to be involved in the speciation of *Ae. markgrafii* (Danilova et al., 2017a). The action of gametocidal genes, which induce chromosome breakage in the gametes lacking them during meiosis (Tsujimoto, 1995), cannot be excluded. Interestingly, gametocidal genes were identified in *Aegilops* species with the C and M genomes on chromosome 3C of *Ae. markgrafii* (Endo and Katayama, 1978) and *Ae. triuncialis* (Endo and Tsunewaki, 1975), on 2C of *Ae. cylindrica* (Endo, 1996) and on chromosome 4M^g^ of *Ae. geniculata* (Kynast et al., 2000).

During the speciation of *Ae. umbellulata, Ae. caudata*, and, to a lesser extent, *Ae. comosa*, the formation of submeta- or acrocentric chromosomes may result in the accumulation of non-recombining regions with loci for adaptive traits (Parisod and Badaeva, 2020). The large non-recombining haplotype blocks were identified recently in sunflower and were associated with adaptive traits, such as seed size, flowering time, and soil fertility (Todesco et al., 2020). The hypothesis on the involvement of rearranged chromosome structure in adaptive radiation of *Ae. markgrafii* and *Ae. umbellulata* relative to *Ae. comosa* seems to be supported by the geographical distribution of these species, with an east-west shift from *Ae. comosa* (occurring mainly in Albania, Balkan Peninsula, and Greece) through *Ae. markgrafii* (abundant in Aegean and Western Turkey) to *Ae. umbellulata* (abundant in Asia Minor, Anatolia, Transcaucasia, and Iran) (Kilian et al., 2011).

Efficient alien gene transfer to wheat requires the ability to screen large pre-breeding populations for the presence of desirable chromosome segments. While recent improvements in the genotyping platforms based on SNP arrays (King et al., 2017) and genotyping-by-sequencing (GBS)-identified SNPs (Edae et al., 2017) are capable to detect alien chromatin transferred into wheat, their use remains expensive for applied research and breeding to genotype thousands of individuals. Moreover, specific SNPs have to be converted to uniplex (KASP) markers for routine work.

In this study, we propose a new approach for developing molecular markers using the sequences of orthologous genes whose physical positions on chromosomes are determined by single-gene FISH. The advantage of this approach is that the determination of marker position does not rely on genetic mapping, which is challenging in *Aegilops* (Edae et al., 2017). Moreover, the positions of markers determined by recombination frequency may differ from the actual physical position (Saintenac et al., 2009). The conservative sequences of cDNA probes can be used for comparative cytogenetics in related species, such as wheat, *Ae. markgrafii* and *A. cristatum* (Danilova et al., 2014, 2017a; Said et al., 2018), as well as in *Ae. comosa* and *Ae. umbellulata*. The results of the sequence similarity search showed that 87.2 and 75% of the cDNAs were located on the same chromosomes in *Ae. comosa* and *Ae. umbellulata*, respectively, as determined by single-gene FISH (Figure 6, Supplementary Data 1).

If DNA sequence data are available for the species of interest, the appropriate level of polymorphism in intronic regions permits the development of molecular markers for defined physical positions on alien chromosomes. In this study, we developed 66 PCR-validated markers for 27 single-gene positions BLAST validated on *Ae. comosa* and 80 markers for 28 cDNA positions BLAST-validated on *Ae. umbellulata*, which were polymorphic to wheat. The fact that most of the markers produced amplicons in *Ae. biuncialis* and in wheat-*Ae. geniculata* chromosome addition lines (Figures 6, 7, Supplementary Data 2) indicates that they will facilitate the introgression of M- and U-genome chromosome segments also from allopolyploid *Aegilops* species into wheat.

Some chromosome regions remain uncovered with markers, such as chromosome arms 1ML, 2MS, and 6MS, or 2US and 7US. After the ordering of chromosomal scaffolds of *Ae. comosa* and *Ae. umbellulata* using the GenomeZipper approach (Mayer et al., 2009), these genomic resources will facilitate the development of additional single-gene FISH probes and molecular markers for targeted chromosomal regions. Moreover, the sequence assemblies obtained from the flow-sorted chromosomes of *Ae. comosa* and *Ae. umbellulata* will facilitate the comparative analysis of repeat landscape and gene content of the M- and U-genome chromosomes, as demonstrated for wheat and for the group 5 chromosomes of *Ae. tauschii* or *Ae. geniculata* (Lucas et al., 2014; Akpinar et al., 2015; Tiwari et al., 2015). These genomic resources will also open the way for visualization, introgression, and cloning of agronomically important *Aegilops* genes in wheat.

The single-gene FISH maps obtained in this study uncovered the long-range chromosome organization of *Ae. comosa* and *Ae. umbellulata*. The comparative cytogenetic analysis demonstrated that most of the M-genome chromosomes are collinear with those of wheat. The U genome is characterized by significant chromosome rearrangements, and several of them are similar to those in *Ae. markgrafii*, indicating that the U genome is more similar to the C genome than to the M genome, not only at the sequence level but at the level of chromosome organization. The physically mapped cDNA probes together with the sequence assemblies of flow-sorted chromosomes permitted the development of PCR markers with precisely determined physical positions on the M- and U-genome chromosomes. The chromosome-specific genomic resources, molecular tools, and single-gene FISH maps will support the introgression of *Aegilops* genes into wheat and their cloning.

## Data Availability Statement

The original contributions presented in the study are publicly available. The generated sequence data of *Ae. comosa* and *Ae. umbellulata* for this study can be found in DRYAD repository at https://doi.org/10.5061/dryad.wpzgmsbk9 and https://doi.org/10.5061/dryad.70rxwdbwc. The cDNA-sequences were developed by the National BioResource Project-Wheat, Japan and were derived from the following resources available in the public domain https://shigen.nig.ac.jp/wheat/komugi/. The cDNA clones and *Ae. comosa* accession MvGB1039 and *Ae. umbellulata* accession AE740/03 together with the other plant genotypes used in the present study are available from the corresponding author upon reasonable request.

## Author Contributions

The work involved collaboration between all authors. BF, JD, IM, and MS conceived the research theme. IM and MS designed the experiments and wrote the first draft of the manuscript. MS carried out the single-gene FISH experiments and analyzed the data. PC carried out flow sorting the chromosomes. IM carried out the purity check. KH and JB sequenced the chromosomes. MA and MM-T assembled the chromosomal contigs. BK carried out the BLASTn search and functional annotation of cDNA-sequences. AF, EG, and LI designed and PCR validated the molecular markers. All authors have contributed to read and approved the manuscript.

## Conflict of Interest

The authors declare that the research was conducted in the absence of any commercial or financial relationships that could be construed as a potential conflict of interest.
