# Supplementary material for: Development of DNA Markers From Physically Mapped Loci in Aegilops comosa and Aegilops umbellulata Using Single-Gene FISH and Chromosome Sequences
Source: Front Plant Sci. 2021 Jun 15;12:689031. doi: 10.3389/fpls.2021.689031 (PMC8240756; doi:10.3389/fpls.2021.689031)
Supplement: Supplementary file 1 [file Data_Sheet_1.zip › Supplementary Figures.PDF]

Supplementary Material

1 Supplementary Figures

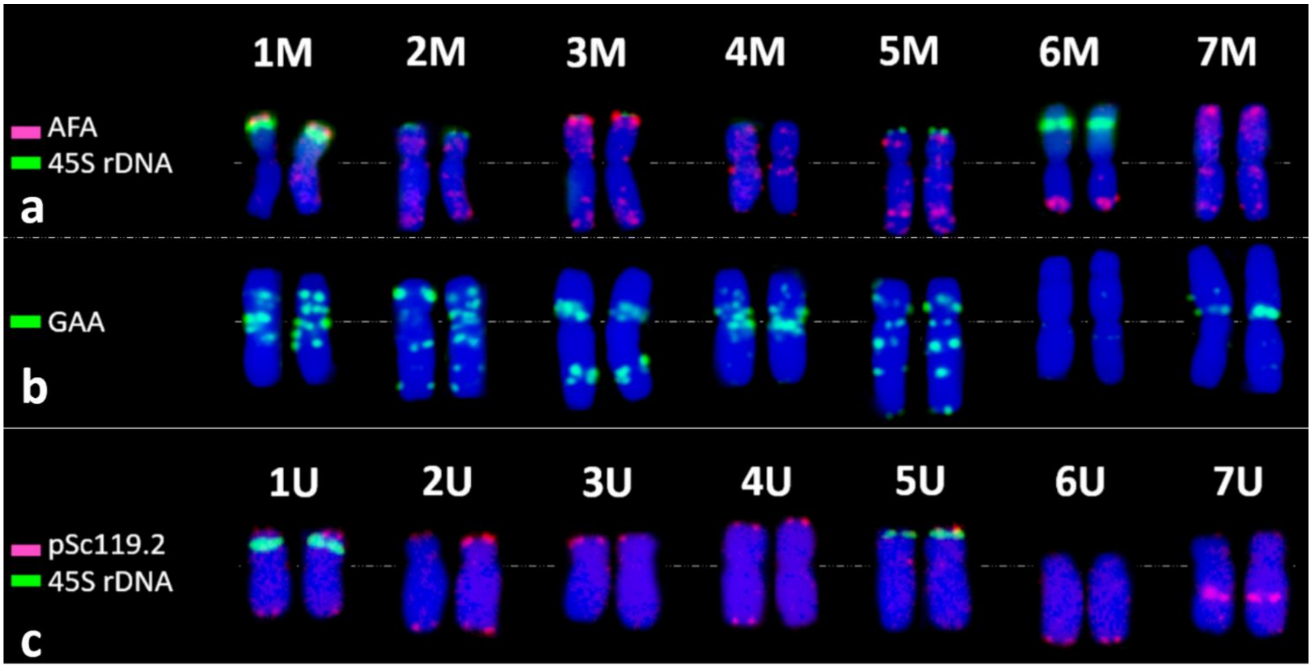

Supplementary Figure 1. FISH on mitotic metaphase chromosomes of *Ae. comosa* and *Ae. umbellulata* with probes for 45S rDNA, *Afa* family repeat, *pSc*119.2 repeat and GAA microsatellites. **a** *Afa* (red) and 45S rDNA (green); **b** GAA microsatellites (green) on chromosomes of *Ae. comosa*; **c** pSc119.2 repeat (red) and 45S rDNA (green) on chromosomes of *Ae. umbellulata*. The chromosomes were counterstained with DAPI (blue).

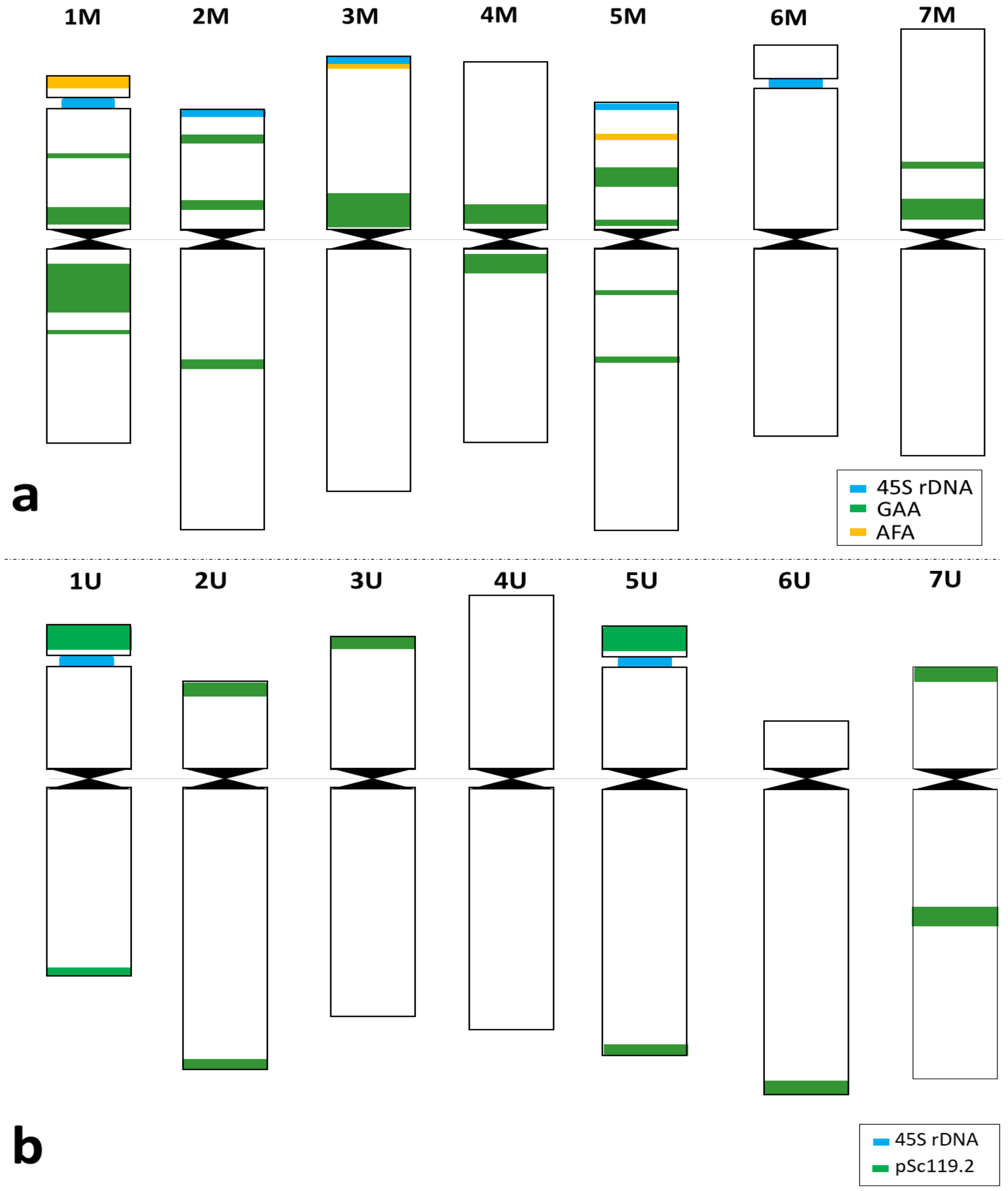

Supplementary Figure 2. Idiogram showing the distribution of five cytogenetic probes identifying *Ae. comosa* and *Ae. umbellulata* individual chromosomes. **a** Pattern of hybridization signals for *Afa* family, 45S rDNA and GAA microsatellites on chromosomes of *Ae. comosa*; **b** Pattern of hybridization signals for pSc119.2 repeat and 45S rDNA on chromosomes of *Ae. umbellulata*. The color schemes (bottom right) show the color of each probe as represented in this idiogram.

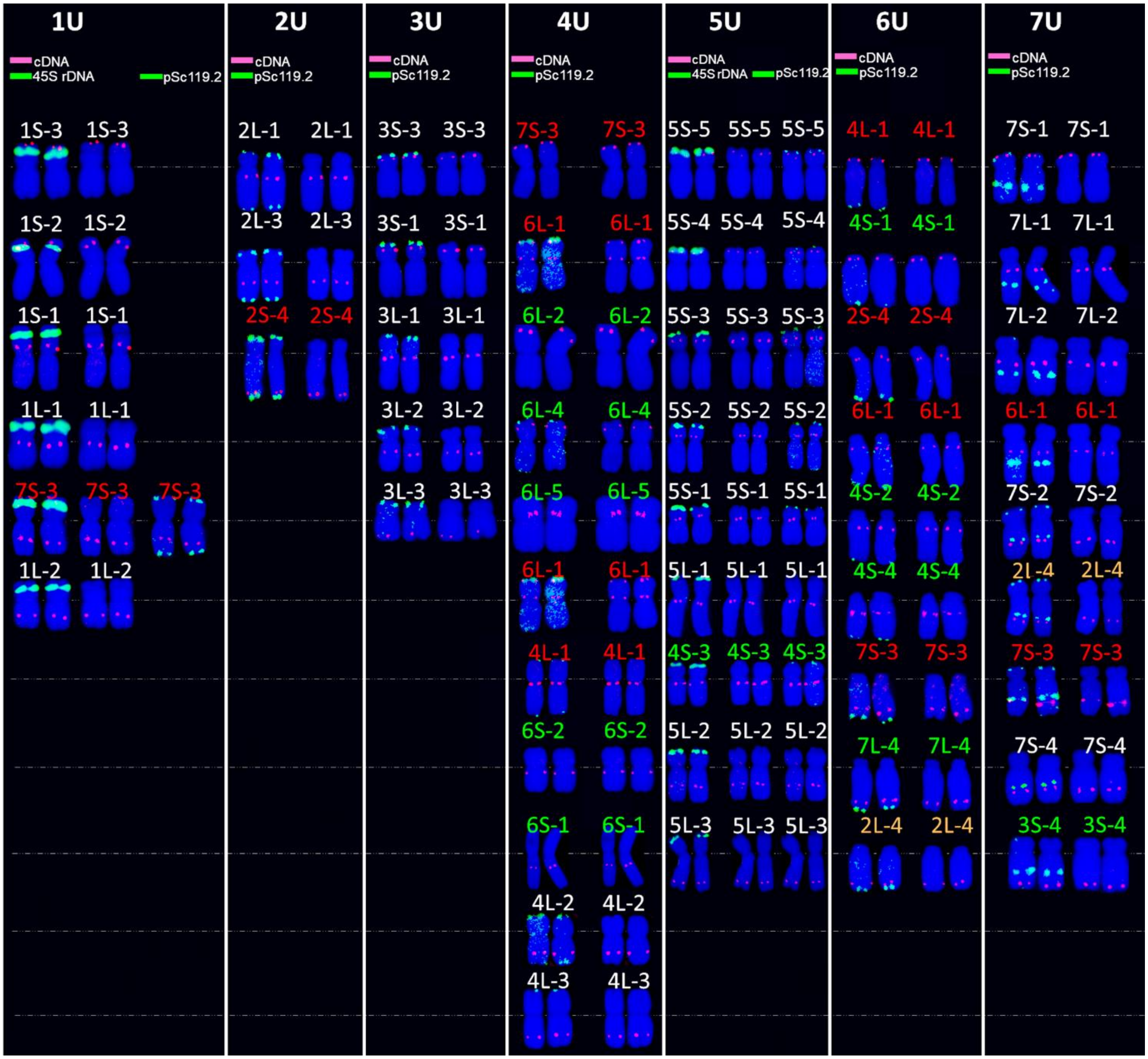

Supplementary Figure 3. FISH on mitotic metaphase chromosomes of *Ae. umbellulata* reveals the distribution of 43 wheat cDNAs (red dots), in addition to 45S rDNA and *pSc*119.2 repeat (both in green). Each chromosome pair (1U-7U) is shown twice or thrice; with the patterns of the repeats in addition to cDNA (left ‘left and right for 1U and 5U’ of the column) and with the cDNA-probes only (right ‘middle for 1U and 5U’ of the column). The names of the cDNA-probes are mentioned above each chromosome pair. The names of cDNA-probes that hybridized to more than one chromosome are highlighted in red. The names of cDNA-probes that hybridized only to a non-homoeologous chromosome are highlighted in green. The names of cDNA-probes that hybridized to two non-homoeologous chromosomes are highlighted in orange.
