# Supplementary material for: Development of DNA Markers From Physically Mapped Loci in Aegilops comosa and Aegilops umbellulata Using Single-Gene FISH and Chromosome Sequences
Source: Front Plant Sci. 2021 Jun 15;12:689031. doi: 10.3389/fpls.2021.689031 (PMC8240756; doi:10.3389/fpls.2021.689031)
Supplement: Supplementary file 1 [file Data_Sheet_1.zip › Supplementary Tables.DOCX]

Supplementary Material

# Supplementary Tables

**Supplementary Table 1.** Chromosome measurements in *Ae. comosa*

| Chromosome | Long arm (L) ± SE  μm | Short arm (S) ± SE  μm | Total length (L+S) = T ± SE  μm | Arm ratio (L/S) | Relative length (T/H) x 100 | Centromeric index (S/T) x100 | Chromosome morphology |
| --- | --- | --- | --- | --- | --- | --- | --- |
| 1M (g) | 3.62 ± 0.05 | 2.91 ± 0.06 | 6.53 ± 0.07 | 1.24 | 12.78 | 44.56 | M^b^ + Sat^c^ |
| 2M (d) | 5.23 ± 0.02 | 2.35 ± 0.04 | 7.58 ± 0.05 | 2.23 | 14.84 | 31.00 | SM^d^ |
| 3M (a) | 4.53 ± 0.03 | 3.25 ± 0.04 | 7.78 ± 0.05 | 1.39 | 15.23 | 41.77 | M |
| 4M (f) | 3.64 ± 0.05 | 3.15 ± 0.02 | 6.79 ± 0.05 | 1.16 | 13.29 | 46.39 | M |
| 5M (c) | 5.21 ± 0.03 | 2.46 ± 0.03 | 7.67 ± 0.06 | 2.12 | 15.02 | 32.07 | SM |
| 6M (e) | 3.53 ± 0.03 | 3.51 ± 0.02 | 7.04 ± 0.03 | 1.01 | 13.78 | 49.86 | M + Sat |
| 7M (b) | 3.90 ± 0.06 | 3.79 ± 0.04 | 7.69 ± 0.07 | 1.03 | 15.05 | 49.28 | M |
| Total |  |  | 51.08 (H^a^) |  | 100 |  |  |

^a^Total length of the chromosomes in the haploid set

^b^Metacentric chromosome

^c^Satellite chromosome

^d^Sub-metacentric chromosome

**Supplementary Table 2.** Chromosome measurements in *Ae. umbellulata*

| Chromosome | Long arm (L) ± SE  μm | Short arm (S) ± SE  μm | Total length (L+S) = T ± SE  μm | Arm ratio (L/S) | Relative length (T/H) x 100 | Centromeric index (S/T) x100 | Chromosome morphology |
| --- | --- | --- | --- | --- | --- | --- | --- |
| 1U (g) | 3.53 ± 0.04 | 2.78 ± 0.05 | 6.31 ± 0.05 | 1.27 | 12.73 | 44.06 | Mb + Satc |
| 2U (d) | 5.22 ± 0.04 | 1.76 ± 0.04 | 6.98 ± 0.05 | 2.97 | 14.08 | 25.21 | SMd |
| 3U (e) | 4.27 ± 0.04 | 2.54 ± 0.05 | 6.81 ± 0.08 | 1.68 | 13.74 | 37.30 | M |
| 4U (a) | 4.55 ± 0.07 | 3.37 ± 0.04 | 7.92 ± 0.07 | 1.35 | 15.97 | 42.55 | M |
| 5U (b) | 4.93 ± 0.09 | 2.75 ± 0.06 | 7.68 ± 0.09 | 1.79 | 15.49 | 35.81 | SM + Sat |
| 6U (f) | 5.59 ± 0.04 | 1.02 ± 0.06 | 6.61 ± 0.07 | 5.48 | 13.33 | 15.43 | SM |
| 7U (c) | 5.31 ± 0.05 | 1.96 ± 0.06 | 7.27 ± 0.10 | 2.71 | 14.66 | 26.96 | SM |
| Total |  |  | 49.58 (H^a^) |  | 100 |  |  |

^a^Total length of the chromosomes in the haploid set

^b^Metacentric chromosome

^c^Satellite chromosome

^d^Sub-metacentric chromosome

**Supplementary Table 3.** Distribution of 43 wheat cDNAs on chromosomes of *Ae. comosa*

| cDNA from wheat chromosome group No. | cDNA detected on homeologous | cDNA detected on homeologous + non-homeologous | cDNA detected on non-homeologous | Total probes | cDNA detected on 7M Short + Long arm |
| --- | --- | --- | --- | --- | --- |
| 1 | 5 (1S-1, 1S-2, 1S-3, 1L-1, 1L-2) | - | - | 5 | - |
| 2 | 2 (2L-3, 2L-4) | 1 (2S-4) | 1 (2L-1) | 4 | - |
| 3 | 6 (3S-1, 3S-3, 3S-4, 3L-1, 3L-2, 3L-3) | - | - | 6 | - |
| 4 | 6 (4S-1, 4S-2, 4S-4, 4L-2, 4L-3, 4L-4) | - | 1 (4S-3) | 7 | - |
| 5 | 8 (5S-1, 5S-2, 5S-3, 5S-4, 5S-5, 5L-1, 5L-2, 5L-3) | - | - | 8 | - |
| 6 | 6 (6S-1, 6S-2, 6L-1, 6L-2, 6L-4, 6L-5) | - | - | 6 | - |
| 7 | 6 (7S-1, 7S-2, 7S-4, 7L-1, 7L-2, 7L-4) | 1 (7S-3) | - | 7 | 2 (7S-3, 7L-4) |
| Total probes | 39 (90.7%) | 2 (4.65%) | 2 (4.65%) | **43** | 2 |
| Total loci | 40 | 5 | 2 | **47** | 4 |

**Supplementary Table 4.** Distribution of 43 wheat cDNAs on chromosomes of *Ae. umbellulata*

| cDNA from wheat chromosome group No. | cDNA detected on homeologous | cDNA detected on homeologous + non-homeologous | cDNA detected on non-homeologous | cDNA detected on double non-homeologous | Total probes |
| --- | --- | --- | --- | --- | --- |
| 1 | 5 (1S-1, 1S-2, 1S-3, 1L-1, 1L-2) | - | - | - | 5 |
| 2 | 2 (2L-1, 2L-3) | 1 (2S-4) | - | 1 (2L-4) | 4 |
| 3 | 5 (3S-1, 3S-3, 3L-1, 3L-2, 3L-3) | - | 1 (3S-4) | - | 6 |
| 4 | 2 (4L-3, 4L-4) | 1 (4L-2) | 4 (4S-1, 4S-2, 4S-3, 4S-4) | - | 7 |
| 5 | 8 (5S-1, 5S-2, 5S-3, 5S-4, 5S-5, 5L-1, 5L-2, 5L-3) | - | - | - | 8 |
| 6 | - | 1 (6L-1) | 5 (6S-1, 6S-2, 6L-2, 6L-4, 6L-5) | - | 6 |
| 7 | 5 (7S-1, 7S-2, 7S-4, 7L-1, 7L-2) | 1 (7S-3) | 1 (7L-4) | - | 7 |
| Total probes | 27 (62.8%) | 4 (9.3%) | 11 (25.6%) | 1 (2.3%) | **43** |
| Total loci | 27 | 12 | 11 | 2 | **52** |

**Supplementary Table 5.** **Table S5.** Purity in chromosome fractions flow-sorted from *Ae. comosa* MvGB1039 and DNA yield

| Chr. | % of the genome | Purity (%) | DNA yield (ng) |
| --- | --- | --- | --- |
| 1M | 12.78 | 83.0 | 45.0 |
| 2M | 14.84 | 80.1 | 46.6 |
| 3M | 15.23 | 97.1 | 94.0 |
| 4M | 13.29 | 72.1 | 57.3 |
| 5M | 15.02 | 86.5 | 44.9 |
| 6M | 13.78 | 98.0 | 90.7 |
| 7M | 15.05 | 89.3 | 37.8 |
| Mean | 14.28 | 86.6 | 59.5 |

**Supplementary Table 6.** Markers designed using selected wheat cDNA sequences and validated by PCR on DNA templates from wheat Mv9kr1, *Ae. comosa*, *Ae. umbellulata*, *Ae. biuncialis*, wheat-*Ae. biuncialis* amphiploid, Chinese Spring-*Ae. comosa*-, Chinese Spring-*Ae. geniculata*- and Chinese Spring-*Ae. umbellulata* addition lines

| Wheat chromosome  group | cDNAs/chromosome group | M-genome based markers | | | Randomly selected for PCR-validation on M genome addition lines | U-genome based markers | | | Randomly selected for PCR-validation on U genome addition lines |
| --- | --- | --- | --- | --- | --- | --- | --- | --- | --- |
|  |  | Markers designed | Wheat/*Aegilops* presence/absence polymorphism | Wheat/*Aegilops* size polymorphism |  | Markers designed | Wheat/*Aegilops* presence/absence polymorphism | Wheat/*Aegilops* size polymorphism |  |
| 1 | 5 | 16 | 2 | 5 | 2 | 15 | 3 | 3 | 4 |
| 2 | 4 | 13 | 1 | 4 | 2 | 12 | - | 2 | 2 |
| 3 | 6 | 22 | 8 | 6 | 4 | 25 | 12 | 3 | 7 |
| 4 | 7 | 21 | - | 15 | 6 | 21 | 3 | 9 | 7 |
| 5 | 9 | 28 | - | 8 | 6 | 27 | 9 | 7 | 8 |
| 6 | 6 | 15 | 5 | 6 | 5 | 16 | 4 | 6 | 2 |
| 7 | 7 | 21 | - | 8 | 3 | 22 | 6 | 7 | 4 |
| **Total** | **44** | **136** | **16** | **52** | **28** | **138** | **37** | **37** | **34** |

**Supplementary Table 7.** Localization of cDNAs by FISH on chromosomes of *Ae. comosa* and *Ae. umbellulata*

| *Aegilops* chromosome | FISH probe order on *Aegilops* chromosomes | Wheat FISH probe name | | Average distance Mean (μm) from the centromere in *Aegilops* | ± SE | Fraction length from the centromere in *Aegilops* | ± SE | FLcDNA, KOMUGI database | cDNA probe length, bp |
| --- | --- | --- | --- | --- | --- | --- | --- | --- | --- |
| 1M | 1MS-3 | | 1S-3 | 2.86 | 0.05 | 0.98 | 0.03 | tplb0048d21 | 3487 |
|  | 1MS-2 | | 1S-2 | 2.64 | 0.05 | 0.90 | 0.02 | AK332649 | 2860 |
|  | 1MS-1 | | 1S-1 | 0.72 | 0.04 | 0.25 | 0.01 | AK333586 | 3522 |
|  | 1ML-1 | | 1L-1 | 0.70 | 0.03 | 0.19 | 0.01 | tplb0013a02 | 5094 |
|  | 1ML-2 | | 2S-4 | 1.12 | 0.04 | 0.31 | 0.01 | tplb0012l12 | 4143 |
|  | 1ML-3 | | 1L-2 | 2.90 | 0.06 | 0.80 | 0.02 | tplb0029f23 | 3113 |
|  |  | |  |  |  |  |  |  |  |
| 2M | 2ML-1 | | 2L-3 | 1.82 | 0.04 | 0.35 | 0.01 | tplb0004a16 | 3841 |
|  | 2ML-2 | | 2L-4 | 4.30 | 0.06 | 0.82 | 0.01 | AK331687 | 4036 |
|  | 2ML-3 | | 2S-4 | 4.72 | 0.04 | 0.90 | 0.01 | tplb0012l12 | 4143 |
|  |  | |  |  |  |  |  |  |  |
| 3M | 3MS-3 | | 3S-4 | 2.90 | 0.03 | 0.90 | 0.01 | tplb0001g16 | 3127 |
|  | 3MS-2 | | 3S-3 | 1.90 | 0.04 | 0.59 | 0.01 | tplb0004j16 | 4402 |
|  | 3MS-1 | | 3S-1 | 1.24 | 0.02 | 0.38 | 0.01 | tplb0014n06 | 3237 |
|  | 3ML-1 | | 3L-1 | 0.44 | 0.02 | 0.10 | 0.01 | AK336104 | 3860 |
|  | 3ML-2 | | 3L-2 | 1.64 | 0.05 | 0.36 | 0.01 | tplb0045e08 | 3369 |
|  | 3ML-3 | | 3L-3 | 3.06 | 0.07 | 0.67 | 0.02 | AK335612 | 3596 |
|  |  | |  |  |  |  |  |  |  |
| 4M | 4MS-3 | | 4S-4 | 2.74 | 0.05 | 0.87 | 0.02 | tplb0043m19 | 3384 |
|  | 4MS-2 | | 4S-2 | 1.94 | 0.09 | 0.61 | 0.03 | tplb0014k23 | 3488 |
|  | 4MS-1 | | 4S-1 | 0.62 | 0.02 | 0.20 | 0.01 | AK330261 | 3582 |
|  | 4ML-1 | | 4L-1 | 0.94 | 0.07 | 0.26 | 0.02 | AK335837 | 3866 |
|  | 4ML-2 | | 4L-2 | 2.14 | 0.02 | 0.59 | 0.01 | tplb0033b21 | 3024 |
|  | 4ML-3 | | 4L-3 | 2.84 | 0.07 | 0.78 | 0.01 | AK335609 | 4790 |
|  |  | |  |  |  |  |  |  |  |
| 5M | 5MS-5 | | 5S-5 | 2.24 | 0.05 | 0.91 | 0.02 | tplb0027f03 | 2416 |
|  | 5MS-4 | | 5S-4 | 2.04 | 0.02 | 0.83 | 0.01 | tplb0016e11 | 2847 |
|  | 5MS-3 | | 5S-3 | 1.72 | 0.02 | 0.70 | 0.01 | tplb0006h03 | 3807 |
|  | 5MS-2 | | 5S-2 | 0.82 | 0.04 | 0.33 | 0.01 | tplb0002p18 | 3112 |
|  | 5MS-1 | | 5S-1 | 0.40 | 0.03 | 0.16 | 0.01 | tplb0016k09 | 3057 |
|  | 5ML-1 | | 5L-1 | 0.40 | 0.03 | 0.08 | 0.01 | tplb0014l23 | 3737 |
|  | 5ML-2 | | 4S-3 | 0.84 | 0.05 | 0.16 | 0.01 | tplb0013i03 | 4240 |
|  | 5ML-3 | | 5L-2 | 2.34 | 0.02 | 0.45 | 0.01 | AK331808 | 4808 |
|  | 5ML-4 | | 2L-1 | 2.62 | 0.04 | 0.50 | 0.01 | tplb0007l09 | 3165 |
|  | 5ML-5 | | 5L-3 | 5.04 | 0.05 | 0.97 | 0.01 | AK334748 | 5408 |
|  |  | |  |  |  |  |  |  |  |
| 6M | 6MS-2 | | 6S-2 | 1.70 | 0.05 | 0.48 | 0.02 | tplb0006a09 | 3685 |
|  | 6MS-1 | | 6S-1 | 1.52 | 0.06 | 0.43 | 0.02 | tplb0050a13 | 3244 |
|  | 6ML-1 | | 6L-5 | 1.34 | 0.02 | 0.38 | 0.01 | tplb0009a09 | 3283 |
|  | 6ML-2 | | 6L-4 | 2.32 | 0.07 | 0.66 | 0.02 | AK333670 | 4377 |
|  | 6ML-3 | | 6L-2 | 2.64 | 0.05 | 0.75 | 0.02 | AK332077 | 5017 |
|  | 6ML-4 | | 7S-3 | 3.12 | 0.06 | 0.88 | 0.02 | tplb0006n08 | 3254 |
|  | 6ML-5 | | 6L-1 | 3.28 | 0.07 | 0.93 | 0.03 | tplb0016o11 | 2658 |
|  |  | |  |  |  |  |  |  |  |
| 7M | 7MS-5 | | 7S-4 | 3.28 | 0.06 | 0.86 | 0.02 | tplb0015e09 | 3640 |
|  | 7MS-4 | | 7S-3 | 3.12 | 0.06 | 0.82 | 0.01 | tplb0006n08 | 3254 |
|  | 7MS-3 | | 7S-2 | 2.34 | 0.05 | 0.62 | 0.01 | tplb0021a05 | 2889 |
|  | 7MS-2 | | 7S-1 | 1.74 | 0.02 | 0.46 | 0.01 | AK334430 | 4404 |
|  | 7MS-1 | | 7L-4 | 0.42 | 0.04 | 0.11 | 0.01 | tplb0007o14 | 3957 |
|  | 7ML-1 | | 7L-1 | 0.34 | 0.02 | 0.09 | 0.01 | tplb0013b07 | 3360 |
|  | 7ML-2 | | 7L-2 | 1.34 | 0.05 | 0.34 | 0.01 | tplb0061d08 | 3147 |
|  | 7ML-3 | | 7S-3 | 3.42 | 0.06 | 0.88 | 0.02 | tplb0006n08 | 3254 |
|  | 7ML-4 | | 7L-4 | 3.74 | 0.05 | 0.96 | 0.01 | tplb0007o14 | 3957 |
|  |  | |  |  |  |  |  |  |  |
| 1U | 1US-3 | | 1S-3 | 2.58 | 0.04 | 0.93 | 0.02 | tplb0048d21 | 3487 |
|  | 1US-2 | | 1S-2 | 2.32 | 0.02 | 0.84 | 0.02 | AK332649 | 2860 |
|  | 1US-1 | | 1S-1 | 0.70 | 0.03 | 0.25 | 0.01 | AK333586 | 3522 |
|  | 1UL-1 | | 1L-1 | 0.72 | 0.02 | 0.20 | 0.01 | tplb0013a02 | 5094 |
|  | 1UL-2 | | 7S-3 | 1.44 | 0.05 | 0.41 | 0.02 | tplb0006n08 | 3254 |
|  | 1UL-3 | | 1L-2 | 1.86 | 0.02 | 0.53 | 0.01 | tplb0029f23 | 3113 |
|  |  | |  |  |  |  |  |  |  |
| 2U | 2UL-1 | | 2L-1 | 1.22 | 0.05 | 0.23 | 0.01 | tplb0007l09 | 3165 |
|  | 2UL-2 | | 2L-3 | 2.30 | 0.06 | 0.44 | 0.02 | tplb0004a16 | 3841 |
|  | 2UL-3 | | 2S-4 | 4.58 | 0.04 | 0.88 | 0.01 | AK331687 | 4036 |
|  |  | |  |  |  |  |  |  |  |
| 3U | 3US-2 | | 3S-3 | 2.14 | 0.07 | 0.85 | 0.04 | tplb0004j16 | 4402 |
|  | 3US-1 | | 3S-1 | 1.62 | 0.04 | 0.64 | 0.02 | tplb0014n06 | 3237 |
|  | 3UL-1 | | 3L-1 | 0.80 | 0.03 | 0.19 | 0.01 | AK336104 | 3860 |
|  | 3UL-2 | | 3L-2 | 1.82 | 0.06 | 0.43 | 0.01 | tplb0045e08 | 3369 |
|  | 3UL-3 | | 3L-3 | 2.84 | 0.05 | 0.66 | 0.01 | AK335612 | 3596 |
|  |  | |  |  |  |  |  |  |  |
| 4U | 4US-5 | | 7S-3 | 3.14 | 0.07 | 0.94 | 0.03 | tplb0006n08 | 3254 |
|  | 4US-4 | | 6L-1 | 2.94 | 0.02 | 0.88 | 0.01 | tplb0016o11 | 2658 |
|  | 4US-3 | | 6L-2 | 2.42 | 0.04 | 0.72 | 0.02 | AK332077 | 5017 |
|  | 4US-2 | | 6L-4 | 1.92 | 0.06 | 0.57 | 0.01 | AK333670 | 4377 |
|  | 4US-1 | | 6L-5 | 0.54 | 0.02 | 0.16 | 0.01 | tplb0009a09 | 3283 |
|  | 4UL-1 | | 4L-1 | 0.34 | 0.02 | 0.08 | 0.01 | AK335837 | 3866 |
|  | 4UL-2 | | 6S-2 | 0.82 | 0.04 | 0.18 | 0.01 | tplb0006a09 | 3685 |
|  | 4UL-3 | | 6S-1 | 1.24 | 0.02 | 0.27 | 0.00 | tplb0050a13 | 3244 |
|  | 4UL-4 | | 4L-2 | 2.64 | 0.04 | 0.58 | 0.01 | tplb0033b21 | 3024 |
|  | 4UL-5 | | 4L-3 | 3.04 | 0.05 | 0.67 | 0.02 | AK335609 | 4790 |
|  |  | |  |  |  |  |  |  |  |
| 5U | 5US-5 | | 5S-5 | 1.80 | 0.05 | 0.66 | 0.02 | tplb0027f03 | 2416 |
|  | 5US-4 | | 5S-4 | 1.52 | 0.07 | 0.55 | 0.02 | tplb0016e11 | 2847 |
|  | 5US-3 | | 5S-3 | 1.12 | 0.06 | 0.41 | 0.03 | tplb0006h03 | 3807 |
|  | 5US-2 | | 5S-2 | 0.62 | 0.02 | 0.23 | 0.01 | tplb0002p18 | 3112 |
|  | 5US-1 | | 5S-1 | 0.26 | 0.02 | 0.10 | 0.01 | tplb0016k09 | 3057 |
|  | 5UL-1 | | 5L-1 | 0.74 | 0.04 | 0.15 | 0.01 | tplb0014l23 | 3737 |
|  | 5UL-2 | | 4S-3 | 1.24 | 0.05 | 0.25 | 0.01 | tplb0013i03 | 4240 |
|  | 5UL-3 | | 5L-2 | 2.54 | 0.04 | 0.52 | 0.02 | AK331808 | 4808 |
|  | 5UL-4 | | 5L-3 | 3.66 | 0.09 | 0.74 | 0.02 | AK334748 | 5408 |
|  |  | |  |  |  |  |  |  |  |
| 6U | 6US-2 | | 4L-1 | 0.84 | 0.07 | 0.83 | 0.07 | AK335837 | 3866 |
|  | 6US-1 | | 4S-1 | 0.50 | 0.03 | 0.49 | 0.03 | AK330261 | 3582 |
|  | 6UL-1 | | 2S-4 | 0.84 | 0.07 | 0.15 | 0.01 | tplb0012l12 | 4143 |
|  | 6UL-2 | | 6L-1 | 1.08 | 0.07 | 0.19 | 0.01 | tplb0016o11 | 2658 |
|  | 6UL-3 | | 4S-2 | 1.64 | 0.02 | 0.29 | 0.01 | tplb0014k23 | 3488 |
|  | 6UL-4 | | 4S-4 | 2.32 | 0.05 | 0.41 | 0.01 | tplb0043m19 | 3384 |
|  | 6UL-5 | | 7S-3 | 3.74 | 0.08 | 0.67 | 0.02 | tplb0006n08 | 3254 |
|  | 6UL-6 | | 7L-4 | 3.98 | 0.13 | 0.71 | 0.02 | tplb0007o14 | 3957 |
|  | 6UL-7 | | 2L-4 | 4.28 | 0.08 | 0.76 | 0.01 | AK331687 | 4036 |
|  |  | |  |  |  |  |  |  |  |
| 7U | 7US-1 | | 7S-1 | 1.90 | 0.04 | 0.97 | 0.03 | AK334430 | 4404 |
|  | 7UL-1 | | 7L-1 | 0.54 | 0.02 | 0.10 | 0.00 | tplb0013b07 | 3360 |
|  | 7UL-2 | | 7L-2 | 1.22 | 0.04 | 0.23 | 0.01 | tplb0061d08 | 3147 |
|  | 7UL-3 | | 6L-1 | 1.88 | 0.06 | 0.35 | 0.01 | tplb0016o11 | 2658 |
|  | 7UL-4 | | 7S-2 | 2.86 | 0.08 | 0.54 | 0.02 | tplb0021a05 | 2889 |
|  | 7UL-5 | | 2L-4 | 3.14 | 0.07 | 0.59 | 0.02 | AK331687 | 4036 |
|  | 7UL-6 | | 7S-3 | 3.72 | 0.04 | 0.70 | 0.01 | tplb0006n08 | 3254 |
|  | 7UL-7 | | 7S-4 | 4.03 | 0.09 | 0.75 | 0.02 | tplb0015e09 | 3640 |
|  | 7UL-8 | | 3S-4 | 4.62 | 0.06 | 0.87 | 0.01 | tplb0001g16 | 3127 |
